# Design of tables for the presentation and communication of data in ecological and evolutionary biology

**DOI:** 10.1002/ece3.10062

**Published:** 2023-07-14

**Authors:** Miriam Remshard, Simon A. Queenborough

**Affiliations:** ^1^ Yale School of the Environment Yale University New Haven CT USA

**Keywords:** alignment, data, graphic design, perception, tables, significance, statistics

## Abstract

Tables and charts have long been seen as effective ways to convey data. Much attention has been focused on improving charts, following ideas of human perception and brain function. Tables can also be viewed as two‐dimensional representations of data; yet, it is only fairly recently that we have begun to apply principles of design that aid the communication of information between the author and reader. In this study, we collated guidelines for the design of data and statistical tables. These guidelines fall under three principles: aiding comparisons, reducing visual clutter, and increasing readability. We surveyed tables published in recent issues of 43 journals in the fields of ecology and evolutionary biology for their adherence to these three principles, as well as author guidelines on journal publisher websites. We found that most of the over 1000 tables we sampled had no heavy grid lines and little visual clutter. They were also easy to read, with clear headers and horizontal orientation. However, most tables did not aid the vertical comparison of numeric data. We suggest that authors could improve their tables by the right‐flush alignment of numeric columns typeset with a tabular font, clearly identify statistical significance, and use clear titles and captions. Journal publishers could easily implement these formatting guidelines when typesetting manuscripts.

## Graphical abstract

Tables are two‐dimensional representations of data, yet it is only recently that we have begun to apply principles of design that aid the communication of information between the author and reader. We surveyed >1000 tables published in recent issues of 43 journals in the field of ecology and evolution and found that most were easy to read with no heavy gridlines and little visual clutter. We suggest that authors and publishers could improve their tables by the right‐flush alignment of numeric columns typeset with a tabular font, clearly identify statistical significance, and use clear titles and captions.

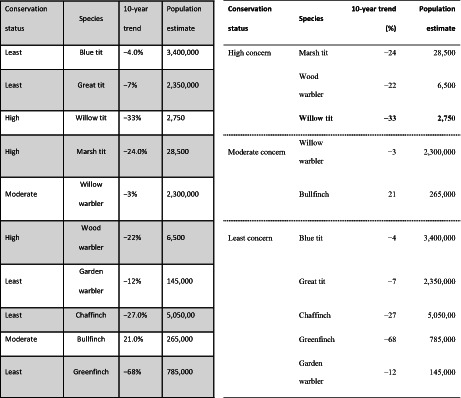

## 1 INTRODUCTION

The idea that we should “Let the data speak for themselves,” is often used by scientists across various disciplines to justify the conclusions they have drawn from their research (e.g., Datta et al., 2021; Effiong & Iriabije, 2018; Yarbrough et al., 2017). This idea is especially prevalent when it comes to tables of data and statistical summaries (Gelman, 2011a). Tables can serve various functions, such as analysis, monitoring, and planning. When included in research papers, tables generally act as a way for authors to provide readers with useful and consequential insight (cf. Few, 2004). Tables are often used to provide summaries of data, statistical results, or descriptions. However, critiques of the commonplace use of tables for displaying research results (e.g., Gelman, 2011a, 2011b; Gelman et al., 2002), as well as calls for improving the design of tables (e.g., Few, 2004; Schwabish, 2020), suggest that presenting numerical values is not as easy as creating a table and letting the data speak. In other words, tables do not inherently communicate. More often, table design can hinder the understanding of data. A better approach, whereby authors design tables to guide the reader and support their interpretations and conclusions, is offered here.

Tables and charts can both be used to quickly and visually present data to an audience or reader. Whether to use a table or a chart to display data will depend on one's goals. Charts encode data values as position, length, size, or color, and support readers when making comparisons, predictions, or perceiving patterns and trends (Coll et al., 1991; Spence & Lewandowsky, 1991). In contrast, there is good evidence that tables are advantageous when the purpose is to extract specific information, precise numerical values, or ranks (cf., Coll et al., 1994; Meyer et al., 1999; Muth, 2019). However, tables are also a visualization to some degree. Tables present the numerical values themselves, and the length (i.e., place value) of these typeset numbers provides visual cues as to their size. Despite suggestions that tables should be used solely to present absolute numbers (cf. Gelman et al., 2002), tables can be designed so as to aid comparisons within their two‐dimensional structure (Few, 2004; Wong, 2010).

There has been an explosion of information and guidelines for the design and presentation of graphs, charts, and figures for business and science since Cleveland (1985). However, table design has been largely ignored (e.g., only 4 of 160 pages in Wong, 2010; 2 of 371 in Wilke, 2019; but see Ryder, 1986, 1995). More hopefully, the design of tables has been the focus of two recent best‐practice guides. Few (2004) focused on table design for the quick and accurate derivation of quantitative business information. Schwabish (2020) described commonly observed flaws in tables in public policy journals and provided suggestions to improve these tables to more effectively communicate their content. Other authors have made recommendations as interest in the design of tables has increased (Dougherty & Ilyankou, 2020; Knaflic, 2015; Muth, 2019, 2022; Schwabish, 2021; Velez, 2020; Wilke, 2019; Wong, 2010). A summary of these current guidelines would be of help to authors and publishers.

The use of tables has been reviewed in various disciplines, largely statistics (Gelman, 2011b; Wainer, 1997), but also public policy (Schwabish, 2020), business (Few, 2004), medicine (Boers, 2018; Schriger et al., 2006), and consumer reports (Vaiana & McGlynn, 2002). We are not aware of such a review paper in the field of ecology and evolution (Greenacre (2016) focused on the content, rather than design, of tables published in ecology journals). In this paper, we begin by collating current best‐practice guidelines for table design. We then examine how well these guidelines are met in recent issues of 43 ecology and evolution journals.

## 2 PRINCIPLES OF TABLE DESIGN

Before reflecting on the quality of tables published in current ecology and evolution journals, it is important to identify principles for table design. To this end, we reviewed the existing academic, business, and gray literature (including reports and websites) to compile the relevant principles and specific guidelines into a single comprehensive resource (Appendix S1). Following these guidelines will likely improve a table in varying ways, as they each fulfill different functions. We have grouped these table design guidelines under the following three main purposes: whether they aid comparisons, reduce visual clutter, or increase readability (Box 1, Table 1).

BOX 1 **Checklist of guidelines for designing tables**

- Aid comparisons
  1. Left‐flush align text and headers
  2. Right‐flush align numbers and their headers
  3. Use the same, appropriate level of precision
  4. Use a tabular font for numeric columns
  5. Use long format (rather than wide format) and white space between rows and columns to guide readers
  6. Include visualizations if suitable
- Reduce visual clutter
  1. Avoid heavy grid lines
  2. Remove unit repetition within cells
  3. Group similar data
- Increase readability
  1. Ensure that headers stand out from the body
  2. Highlight outliers and/or statistical significance
  3. Use active, concise titles
  4. Orient tables horizontally

**TABLE 1:**
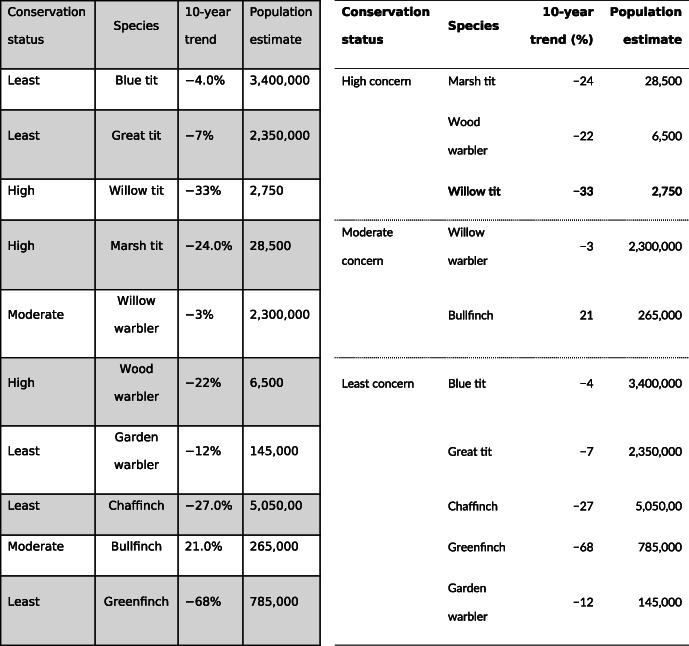
Illustration of worse (left) and better (right) design of tables. In comparison to the worse design, the formatting in the better table aids comparisons (left‐flush aligned text, right‐flush aligned numbers, with constant precision, in a tabular font), reduces visual clutter (no heavy grid lines, removed unit repetition, and grouped the data), and increases readability (clear headers, highlighting outliers). See Appendix S2 for more examples. Data from Burns et al. (2020).

*Note*: The bold font highlights the species of focus.

### 2.1 Aid comparisons

A strength of tables is that they allow the reader to make comparisons along two dimensions. One could even view tables as elementary horizontal bar charts, where the typeset numbers correspond to the bars (Gelman, 2011a). Using the standard decimal system of positional notation, numbers grow in length from right to left as they increase in the size of the value they represent. As such, the main purpose of a table, after presenting the actual numbers themselves, is to help the reader compare these numbers as their eye moves up and down columns and horizontally along rows. To support this goal, there are several table design principles that aid readers in making the desired comparisons of numeric data.

#### 2.1.1 Left‐flush align text and headers

Alignment is important when presenting different types of data and should follow the natural direction of reading and writing. In languages where text is read and written from left to right (such as English), left‐flush alignment of text and headers enhances the readability of these columns.

#### 2.1.2 Right‐flush align numbers and their headers

In contrast to text, numbers increase in size from right to left. It is therefore much easier to compare values if numerical columns and their corresponding headers are right‐flush aligned.

#### 2.1.3 Use the same, appropriate level of precision

In addition to a right‐flush alignment, all the cells of a column must have the same level of precision otherwise their place values will not be consistent. Equal precision may require rounding and/or adding zeroes after the decimal point. The use of commas can help group sets of three place values. Large values can be rounded to the nearest thousand, million, etc. to avoid unnecessary repetition of zeros (as long as this is indicated in the header).

#### 2.1.4 Use a tabular font for numeric columns

The vertical alignment of place value is ensured by using a tabular font. In contrast to a proportional font (where each number takes up space in proportion to its size), in a tabular font each number has the same width. Thus, in a tabular font, the number 111.1 will have the same length as 888.8, and each place value will be aligned vertically. Tabular fonts include Lato, Noto Sans, Open Sans, Roboto, and Source Sans Pro.

#### 2.1.5 Use long format (rather than wide format) and white space between rows and columns to guide readers

When thinking about the layout of a table, consider that data are often easier to compare when presented vertically rather than horizontally (see #2, 3, and 4 above). What comparisons do you want the reader to make? Do you need to rotate your table to aid those? Use the potential collection of new data to inform this decision: is new data added as additional rows below the existing data or do you need to create new columns, suggesting that a table redesign is needed?

The spacing between rows and columns further influences how a table is read. To support vertical comparisons, increasing the space between columns and reducing it between rows encourages readers to view the table from top to bottom. In contrast, increasing space between rows promotes the horizontal viewing of tables. Furthermore, authors should bear in mind that most tables in printed or typeset articles will be set to fit either a single or double column width. Even wider tables are often rotated by 90° (see 2.3.5, below).

#### 2.1.6 Include visualizations if suitable

Many recent authors have suggested combining charts and their ability to show patterns in data with the precision of tables. For example, icons, bars, or sparklines can be included as separate columns. Icons can encode qualitative information, such as nation‐state (e.g., flags) or order within Animalia (e.g., drawings of stereotypical animals). Icons can also encode quantitative data summarized as a category, for example using arrows to highlight the direction of a trend, or a positive or negative result. Bars can provide a clear visualization of magnitude, using length to encode value as in a traditional horizontal bar chart. Sparklines (tiny line charts, often without axes or coordinates) can be used to show the general pattern of variation or trends over time.

Finally, heatmaps add visual elements to a table, without the use of additional columns. Encoding the background of each cell with a different hue or saturation of color depending on the data value, heatmaps can display general patterns in the data and are often suitable for presenting frequency data. As always when using color, take care to follow best‐practice guidelines for encoding data and human perception (Datawrapper, 2021; Light & Bartlein, 2011).

### 2.2 Reduce visual clutter

Regardless of their precise function, all tables present a lot of information in a dense manner. To support readers in quickly extracting the key information when looking at a table, many authors have argued that it is important to remove any visual clutter. There are three main ways this can be achieved.

#### 2.2.1 Avoid heavy grid lines

Including every cell border makes the table more visually busy and can distract readers from the data. In many cases, only three horizontal lines are needed in a table, one to delineate the top, one the bottom, and a third to identify the header (see 2.3.1, below). In most tables, columns can be delineated by white space.

#### 2.2.2 Remove unit repetition within cells

Using the title, column header, or first row of a table to establish relevant units avoids visually cluttering the table by including the units in every cell.

#### 2.2.3 Group similar data

In addition, grouping similar data, for example, by listing relevant labels only once or by using spanner heads, decreases how cluttered a table may appear.

### 2.3 Increase readability

While the principles described to reduce visual clutter involved removing extraneous information to help focus the reader's attention on the relevant information, the following guidelines suggest how to modify elements that cannot be removed.

#### 2.3.1 Ensure that headers stand out from the body

Use lines and/or boldface type to offset headers clearly from the body of the table.

#### 2.3.2 Highlight outliers

Many readers examine tables to identify the most important values, so highlighting outliers can serve to quicken this search.

#### 2.3.3 Highlight statistical significance

Values that attain statistical significance are one form of “outlier” meriting its own guideline that we could not find previously identified by others. Authors highlight significant values of statistical tables (including boldface font, asterisks, and superscript letters) to make it easier for the reader to quickly understand the results and conclusions. In almost all cases, these elements are added within existing numerical columns and the actual p‐values are not displayed; rather an indication of significance is added to the coefficient estimates. These added elements then tend to disrupt the right‐flush alignment of the column, inhibiting comparisons. A better approach might be to add additional elements to the left of each numeric value, or use a separate column for statistical significance.

#### 2.3.4 Use active, concise titles

What is the message or take‐away result you are conveying with the table? By phrasing titles in an active and concise manner, table captions and titles can be used to guide the reader to focus on what the author considers important and support them in easily understanding the desired key message.

#### 2.3.5 Orient tables horizontally

The orientation of text affects readability. A horizontal orientation is easiest to read. Consequently, tables should also be printed horizontally.

## 3 METHODS

Once we had established the above principles of table design, we assessed their use in issues of 43 widely read ecology and evolution journals (Appendix S2). Between January and July 2022, we reviewed the tables in the most recent issue published by these journals. For journals without issues (such as *Annual Review of Ecology, Evolution, and Systematics*, or *Biological Conservation*), we examined the tables in issues published in a single month or in the entire most recent volume if few papers were published in that journal on a monthly basis. We reviewed only articles in a traditionally typeset format and published as a PDF or in print. We did not examine the tables in online versions of articles.

Having identified all tables for review, we assessed whether these tables followed the above‐described best practice principles for table design and, if not, we noted the way in which these tables failed to meet the outlined guidelines. We initially both reviewed the same 10 tables to ensure that we agreed in our assessment of whether these tables followed each of the principles. Having ensured agreement on how to classify tables, we proceeded to review all subsequent journals individually while resolving any uncertainties collaboratively. These preliminary table evaluations also showed that assessing whether tables used long format or a tabular font was hard to evaluate objectively without knowing the data or the font used. Therefore, we did not systematically review the extent to which these two guidelines were adhered to.

To examine whether adherence to best‐practice guidelines varies depending on the kind of information displayed in the table, we delineated three different types of tables. Statistical tables could range from correlation matrices to showing summary results of various statistical tests (e.g., mean, 25th and 75th percentiles, *t/F/P* values, degrees of freedom) to providing different kinds of information on models (e.g., probability at which a model is chosen for the given data, model predictions, model fitting times, model convergence rates). Text tables contained little or no numerical data. They often served to provide descriptions of measures/factors/analytical approaches used, or examples to demonstrate groupings/category labels, or summaries of different approaches/criticisms from the literature. Data tables either contained data in its unaggregated (raw) format or in a summarized form (e.g., summarizing the frequency of occurrences or categories of interest).

After reviewing issues in each of the 43 journals, we grouped descriptions of the ways in which tables failed to follow best‐practice guidelines and tabulated how often the above principles were met. We also examined the formatting guidelines for tables provided by each journal. All data cleaning and analyses were carried out in the statistical software environment R 4.2.2 (R Development Core Team, 2022).

## 4 RESULTS

**FIGURE 1:**
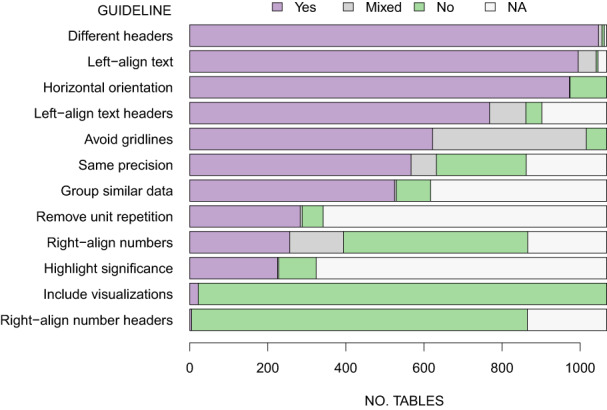
Summary of the number of 1068 tables surveyed for table design guidelines, sampled from papers published in 2022 in 43 ecology and evolution journals. Tables were scored as meeting (Yes), sometimes meeting (Mixed), or not meeting (No) the guidelines. The classification “NA” (Not Applicable) was used if tables did not contain the kind of content relevant to specific guidelines (e.g., text tables contained little or no numerical data, so the principles of removing unit repetition, right‐aligning numbers and highlighting statistical significance did not apply).

In total, we reviewed 1068 tables across all 43 journals (Figure 1). There were twice as many data (448) or statistical (401) tables as text tables (219). The open‐access journals *Ecology & Evolution* and *Neotropical Biodiversity* had by far the most tables (both with >100), double that of the next highest journals such as *Biotropica*, *Biological Conservation*, *Conservation Biology*, *Functional Ecology*, and *Ecosphere* (that had 40–50). The number of papers and tables assessed per journal examined are listed in Appendix S3. Further details of the results are provided in Appendix S4.

### 4.1 Aid comparisons

#### 4.1.1 Left‐flush alignment of text and headers

Almost all (93%, or 995) published tables adhered to the principle of left‐flush alignment of text columns. Of those tables where text columns had a mixed alignment (46), all but one were left‐flush aligned and centered.

Similarly, most (72%, 768) tables had a left‐flush alignment of the headers of text columns. Approximately twice as many tables contained both left‐flush aligned and centered text headers (93) as tables (41) that had only centered text headers.

#### 4.1.2 Right‐flush align numbers and their headers

In contrast to text columns, only 24% (*n* = 256) tables met the recommended right‐flush alignment of numeric columns. However, approximately half the tables we classed as following this principle did not necessarily right‐flush align their numerical columns but were classed as “Yes” because alignment and precision were the same across all values, thereby ensuring easy comparability of values.

Of the many tables (472) that did not follow right‐flush alignment of numeric columns, most (316) used a left‐flush alignment, although centered (65) and decimal‐aligned (59) numbers were also common. Other tables had variable alignment of different numeric columns (84), and others (54) even had variable alignment within columns (often due to different types of data, such as mean and standard deviation, in the same column).

Even more extreme was the lack of adherence to the principle of right‐flush aligning the headers of number columns. Only five tables right‐flush aligned their number headers. Of the 860 tables that did not follow this principle, the majority left‐flush aligned their number heads (629) or centered them (221).

#### 4.1.3 Use the same, appropriate level of precision

Of the tables that contained numerical values (862), most maintained the same level of precision throughout (567). Several tables generally used the same level of precision except for a single value or except for zeroes and ones (65). However, approximately a quarter of tables did not maintain the same level of precision within columns (230).

#### 4.1.4 Include visualizations if suitable

Only 22 of the 1068 tables contained any form of visualization. More than half of these (12) simply used cell shading as a means of visually grouping the table contents. The remaining tables either contained images (4), graphs (2), symbols (1), or they acted as heatmaps (3).

### 4.2 Reduce visual clutter

#### 4.2.1 Avoid heavy grid lines

Most tables (622) did follow the principle of avoiding the use of heavy grid lines. Of those tables that did somehow differentiate different rows or groups, 394 used cell shading (e.g., rows were alternated shaded white and gray or white and blue). Only 52 tables contained grid lines. Of those, 30 had only horizontal grid lines, only one table had only vertical grid lines, and 21 tables contained both types of grid lines.

#### 4.2.2 Remove unit repetition within cells

While many tables (726) did not contain units, those that did largely removed unit repetition (283). The most common approach to meeting this guideline was noting the unit only once in the column header (161). The other two main ways that unit repetition was avoided was by noting the unit in the cells of the first column or in the table title. Only five tables inconsistently avoided unit repetition.

#### 4.2.3 Group similar data

The final principle for reducing visual clutter was also mostly met. While only 88 tables failed to group similar data and four tables did so inconsistently, 525 tables did follow this guideline. Different parts of the table can be grouped. The most common way of grouping information was via the stub (220). This was followed by table spanner grouping (115) and column spanner grouping (111). Four tables did group their data in one of these ways but did not apply another applicable form of grouping the data.

### 4.3 Increase readability

#### 4.3.1 Ensure that headers stand out from the body

This principle was the most consistently followed, with 1047 of the 1068 tables separating headers from the table body in some way. Of these, most used a line (455), or shaded the header cells and used bolded text (387), and some even used both boldface and a line (134).

#### 4.3.2 Highlight outliers

Not all principles that serve to increase readability were followed. Beyond highlighting statistically significant values (see below), not a single table that we reviewed visually distinguished outliers.

#### 4.3.3 Highlight statistical significance

Of the 324 tables that presented *p*‐values or other similar measures, 69% did highlight values that met the chosen alpha threshold for statistical significance (225). Most of these significant values were highlighted via boldface text (104), or they were distinguished using one or more asterisks (60).

#### 4.3.4 Use active, concise titles

Table titles were classed as concise if they contained fewer than 20 words. A total of 408 tables that we reviewed had table titles that were descriptive and not concise, thereby failing to adhere to both components of this best‐practice principle for table design. A total of 643 tables had titles that were concise but not active. However, among 204 of these tables, the title did not appear concise on first glance because the title was combined with the table notes, rather than those appearing below the table.

Four tables had an active title that was nonetheless descriptive, rather than serving to present the key message to readers. Of these only the title of two was also concise. Conversely, three tables had titles phrased as passive messages. Only one of these also had a concise title. Finally, seven tables had titles that served as active messages. However, six of these titles were not concise. Therefore, only one table fully adhered to this guideline and even this one did not appear concise due to combining the table notes and title.

#### 4.3.5 Orient tables horizontally.

This principle was widely followed. Less than 10% of tables were oriented vertically rather than horizontally (94), and one table was oriented horizontally but contained vertical text.

### 4.4 Journal guidelines on formatting tables.

With one exception, all journals reviewed included some guidelines on formatting tables. Slightly more than half of these journal guidelines referenced at least some of the principles for table design discussed above. However, often journal guidelines focused predominantly on where tables should be placed when submitting manuscripts for review; the position, length, and content of table notes or footnotes; the format that tables should be in; and/or the formatting tools that should be used when creating tables, rather than on table design features to support clearer presentation and communication of the data.

Among those journals whose guidelines did touch on some of the content we discuss here, the following guidelines were covered: Avoid heavy grid lines, remove unit repetition within cells, group similar data, right‐flush align numbers, use the same, appropriate level of precision, orient tables horizontally and use active, concise titles. Of these, the ones that were most commonly mentioned were those aimed at reducing visual clutter, with other guidelines only mentioned by one or two journals. Furthermore, the description of these table design guidelines was often very limited (e.g., several journals merely stated that tables should not contain vertical lines or that titles should be “concise”). Occasionally, the journal guidelines on formatting tables were even in conflict with the guidelines we compiled (e.g., advising authors to center columns containing numerical data).

## 5 DISCUSSION

In a survey of over 1000 tables published recently in 43 journals in the field of ecology and evolution, we found varied adherence to principles of table design. Most tables had little visual clutter and no heavy grid lines, and were also easy to read, with clear headers and horizontal orientation. However, most tables did not follow right‐flush alignment of numeric columns, inhibiting the vertical comparison of their values. This issue, along with the identification of statistical significance and the use of titles, are clear areas for improvement.

### 5.1 Table design in ecology and evolution journals

While many statisticians have been at the forefront of thinking about better ways to present data, many of those ideas have resided in the statistical (Ehrenberg, 1978; Gelman, 2011b; Gelman et al., 2002; Ryder, 1986, 1995; Wainer, 1997) or business literature (Few, 2004; Wong, 2010) and are only recently being made widely available (e.g., Dougherty & Ilyankou, 2020; Muth, 2019, 2022).

Recent surveys of the literature in other fields have lamented the lack of high‐quality table design (Boers, 2018; Schriger et al., 2006; Schwabish, 2020). In comparison, we suggest that the design of tables in ecology and evolution may not be as bad, and that some of the guidelines described above are being implemented to some degree. We recommend in future that both publishers and authors focus on aiding comparisons using tabular fonts (now easily available in word‐processing software) and right‐flush alignment of numeric columns.

### 5.2 Practicalities of table design

Both authors and publishers can take simple steps to improve the design of tables published in ecology and evolution journals. Authors can follow the principles we have outlined and provide tables that are designed to aid comparisons, with little visual clutter, and that are easy to read. All these principles are easy to implement in the many software tools available for formatting tables. These tools include spreadsheets, embedding tables in word processors, and specific visualization software (Datawrapper is particularly good for tables). Deep thought about what message the author wants the reader to extract from the table (identical to the process for figures) will help guide what comparisons to facilitate, what groupings to set, and in particular what captions to provide.

However, many formatting decisions are under the full control of publishers. Few publishers provide any guidance at all on tables or their design. A short checklist under the instructions for authors of each journal of the above three principles and 14 guidelines would provide a simple positive step to help authors. Including such a checklist for reviewers could also be helpful. Even better would be for publishers to follow the principles and guidelines themselves, formatting tables consistently to follow the correct alignment of text and numeric columns, and using tabular fonts.

### 5.3 The future of tables and table design.

The use of tables remains a fundamental way to present data and make it widely available. However, there is some conflict between the human readability versus machine readability of tables. Human‐readable tables have much additional formatting and typesetting overlain on the basic structure of rows and columns, which may make them more complex for computers to read. Indeed, in our survey, the few tables that included visualizations were labeled as figures and provided as image files, not as tables.

Resolving these issues will take some thought, but could be straightforward if combined with stricter policies on the archiving of raw data as well as providing the code used to derive these summary and statistical tables from this raw data. Viewing tables within scientific articles to hold a similar function to figures (i.e., supporting the argument rather than being a “data dump”) should also help this endeavor, by encouraging more thought about their need and use.

Finally, we encourage authors to consider the merits of tables and figures and use the most appropriate presentation of their data. Gelman (2011a, 2011b) exhorts authors to consider using figures instead of tables, especially if direct comparisons between several columns are needed (e.g., in the case of model summaries of coefficient estimates, errors, and statistical significance). Aside from the appearance of tables and figures, the most important aspect is, of course, their contents.

## AUTHOR CONTRIBUTIONS

**Miriam Remshard:** Data curation (equal); formal analysis (equal); methodology (equal); visualization (equal); writing – original draft (equal); writing – review and editing (equal). **Simon A. Queenborough:** Conceptualization (lead); data curation (equal); formal analysis (equal); methodology (equal); visualization (equal); writing – original draft (supporting); writing – review and editing (equal).

## CONFLICT OF INTEREST STATEMENT

The authors have no conflicts of interest.

## DATA AVAILABILITY STATMENT

All raw data are archived in DataDryad: https://doi.org/10.5061/dryad.jq2bvq8f3

## Supporting information


Appendix S1

Appendix S2

Appendix S3

Appendix S4

Appendix S5
