# Supplementary material for: Design of tables for the presentation and communication of data in ecological and evolutionary biology
Source: Ecol Evol. 2023 Jul 14;13(7):e10062. doi: 10.1002/ece3.10062 (PMC10346464; doi:10.1002/ece3.10062)
Supplement: Supplementary file 1 — Appendix S1 [file ECE3-13-e10062-s003.pdf]

Supplementary Information 1

Summaries of guidelines on table design identified by key resources

Dougherty, J. & Ilyankou, I. 2020. *Hands-On Data Visualization. Interactive storytelling from spreadsheets to code*. O’Rielly Media Inc.

URL: https://handsondataviz.org/table.html

Summary of Schwabish (2020)

1. Make column headers stand out above the data.
2. Use light shading to separate rows or columns.
3. Left-align text and right-align numbers for easier reading.
4. Avoid repetition by placing labels only in the first row.
5. Group and sort data to highlight meaningful patterns.

When creating cross-tabulations to illustrate data correlations and possible causal relationships:

6. Place the independent variable (the suspected cause) at the top in the column headers, and the dependent variable (the possible effect) on the side for each row.
7. Calculate percentages from raw numbers in a vertical direction going downward, so that each value of the independent variable (the suspected cause) totals 100 percent.

Few, S. 2004. *Show Me The Numbers. Designing tables and graphs to enlighten*. Analytics Press, USA.

Summary of details provided in Ch. 8 Table design.

Columns and rows

Use white space to delineate whenever possible.

If not, use subtle colors or rules.

Avoid grids.

Orientation

Avoid text orientation other than horizontal, left-to-right.

Alignment

Align numbers to right, align decimals as well.

Align dates as needed, but maintain consistency in format.

Align all other text to right.

Number formatting

Comma to left of every three whole-number digits.

Truncate by nearest thousand/million/etc if precision allows.

When negative numbers are enclosed in parentheses, keep aligned with positive numbers.

Date formatting

Months as two-digit number or three character word.

Days as two digits.

Years as two or four digits, consistently.

Number and date precision

Keep to required precision.

Font

Use a legible font,

Use the same font throughout the table.

Use boldface, italics, or color to group or highlight.

Knaflic, C.N. 2015. *Storytelling with Data. A data visualization guide for business professionals*. Wiley, USA.

Use minimal borders and white space to improve the legibility of your table.

Convert table to heatmap.

Velez, A. 2020. *What is a Table?* Storytelling with Data blog

URL: https://www.storytellingwithdata.com/blog/2020/9/24/what-is-a-table

When should you use a table to communicate data?

You have varied data.

Your audience has different needs.

You’re reading line by line.

You’re supplementing the main story.

When shouldn’t you use a table to communicate data?

You are presenting your data live—in person or virtually.

You want to emphasize a pattern or shape in the data.

How can you design a better table?

Clarify horizontal or vertical.

Remove unnecessary borders and shading.

Incorporate a visual element.

Order intentionally.

Use super-category labels.

Align thoughtfully.

Muth, L.C. 2019 *What to consider when creating tables. Dos & don’ts of table design*. Datawrapper blog.

URL: https://blog.datawrapper.de/guide-what-to-consider-when-creating-tables/

When to use tables

Use tables if you want to enable readers to look up specific information. Use charts instead of tables if you want to give an overview of your numerical data or show a pattern.

Use tables if precise numbers are important.

Tables are great for data we’re used to reading.

Tables work better than charts if your readers should compare data in two directions. I

Tables are great for showing ranks.

How to create better tables

Consider narrowing (down) your columns.

Consider structuring your data so that your table has more rows than columns.

Consider grey stripes for long tables with many columns.

Consider adjusting the row height to the number of rows.

Consider pagination for long tables.

Use colors to lead the reader’s eye to interesting information & help them navigate the table

If your table contains information which is more relevant to some readers, but less relevant to others, consider making your table searchable & sortable.

Put some consideration into how your table is sorted.

Consider visualizing your data with bar charts.

Consider visualizing your data with heatmaps.

Consider showing development over time instead of just data from two or three time points.

Muth, L.C. 2022. Which fonts to use for your charts and tables …and how to customize them.

URL: https://blog.datawrapper.de/fonts-for-data-visualization/

Choosing a font

1. Use sans-serif typefaces.

2. Use a font with lining and tabular numbers.

- Lining numbers “line up”: They’re all the same height. Oldstyle numbers go below and above the “line.” They’re common in serif typefaces like Georgia and beautiful to look at in a paragraph, but hard to read in a table.
- Tabular figures are, as the name implies, great for tables. That’s because every number is the same width. It’s easy to see instantly how many figures a number has, because a 124.17 will be the same length as a 680.90, but shorter than a 1,111.17.

3. Use a font with all the symbols you need.

4. Use bold fonts only for emphasis.

5. Avoid very thin fonts.

6. Use neither overly narrow nor overly wide fonts.

Adjusting the font

7. Use text that’s big enough.

8. Use a high-contrast color for most text.

9. Use uppercase text sparingly.

Schwabish, J.A. 2020. Ten guidelines for better tables. *Journal of Benefit-Cost Analysis*, 11, 151–178.

Schwabish, J.A. 2021. *Better Data Visualizations. A guide for scholars, researchers, and wonks*. Columbia University Press, USA.

Ten guidelines for better tables

1. Offset the headers from the body.
2. Use subtle dividers instead of heavy gridlines.
3. Right-align numbers and headers.
4. Left-align text and headers.
5. Select the appropriate level of precision.
6. Guide the reader with space between rows and columns.
7. Remove unit repetition.
8. Highlight outliers.
9. Group similar data and increase white space.
10. Add visualizations when appropriate.

Wilke, C. O. 2019. *Fundamentals of Data Visualization. A primer on making informative and compelling figures*. O'Rielly Media Inc. USA.

URL: https://clauswilke.com/dataviz/figure-titles-captions.html#tables

Rules for formatting tables

1. Do not use vertical lines.
2. Do not use horizontal lines between rows.
3. Text columns should be left aligned.
4. Number columns should be right aligned and should use the same number of decimals digits throughout.
5. Columns containing single characters should be centered.
6. Header fields should be aligned with their data.

Wong, D.M. 2010. *The Wall Street Journal Guide to Information Graphics. The dos and don't of presenting data, facts, and figures*. Norton, New York, NY., USA.

Grid lines

Avoid unhelpful grids

Use thin rules every few rows to guide the reader

Include charts of the main message within tables.

Number alignment and ordering

Never align whole numbers flush left, never align decimals flush left or flush right, never order entries randomly.

Align whole numbers flush right, align decimals on the decimal point, order entries logically.
