# Supplementary material for: Design of tables for the presentation and communication of data in ecological and evolutionary biology
Source: Ecol Evol. 2023 Jul 14;13(7):e10062. doi: 10.1002/ece3.10062 (PMC10346464; doi:10.1002/ece3.10062)
Supplement: Supplementary file 2 — Appendix S2 [file ECE3-13-e10062-s001.pdf]

Supplementary Information 2.

Illustrated guidelines for designing tables

For each guideline, we provide a comparison of a table that does not (above) and a table that does (below) follow the guideline.

(I) Aiding comparison

1. Left-align text and their heads

Species	Conservation status
Cetti's warbler	Least concern
Wood warbler	High concern
Willow warbler	Moderate concern
Sedge warbler	Least concern
Reed warbler	Least concern
Grasshopper warbler	High concern
Garden warbler	Least concern

Species	Conservation status
Cetti's warbler	Least concern
Wood warbler	High concern
Willow warbler	Moderate concern
Sedge warbler	Least concern
Reed warbler	Least concern
Grasshopper warbler	High concern
Garden warbler	Least concern

2. Right-align numbers and their heads

Species	Long-term trend (%)	10-year trend (%)	Survival change (%)	Population estimate
Chaffinch	0	−27	−4.9	5,050,000
Bullfinch	−38	21	−36.0	265,000
Greenfinch	−64	−68	−4.9	785,000

Species	Long-term trend (%)	10-year trend (%)	Survival change (%)	Population estimate
Chaffinch	0	−27	−4.9	5,050,000
Bullfinch	−38	21	−36.0	265,000
Greenfinch	−64	−68	−4.9	785,000

3. Use the same, appropriate level of precision

Species	Survival change (%)	Productivity change (%)
Barn owl	−9.4	6.812
Little owl	−9	43
Tawny owl	5.643	9.6

Species	Survival change (%)	Productivity change (%)
Barn owl	−9.4	6.8
Little owl	−9.0	42.6
Tawny owl	5.6	9.6

4. Include visualizations if suitable

Conservation status	Species	Population estimate
Least concern	Woodpigeon	5,150,000
	Collared dove	810,000
Non-native or occasional visitor	Feral pigeon	465,000
Moderate concern	Stock dove	320,000
High concern	Turtle dove	3,600

Conservation status	Species	Population estimate
Least concern	Woodpigeon	5,150,000
	Collared dove	810,000
Non-native or occasional visitor	Feral pigeon	465,000
Moderate concern	Stock dove	320,000
High concern	Turtle dove	3,600

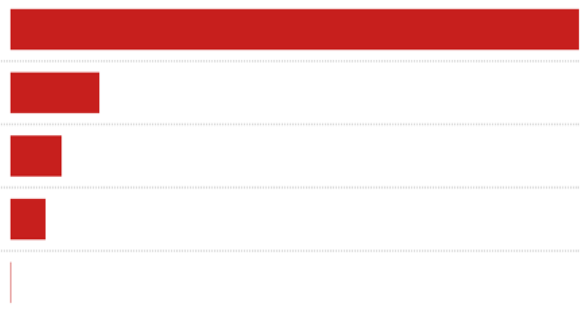

5. Use long format (rather than wide format) and utilize space between rows and columns to guide readers

Species	Blue tit	Great tit	Long-tailed tit	Willow warbler	Sedge warbler	Reed warbler	Chaffinch	Bullfinch	Greenfinch
Long-term trend %	21	79	102	−43	−43	117	0	−38	−64
10-year trend %	−4	−7	0	−3	−3	−4	−27	21	−68
Survival change %	12.6	2.1	17.9	−26.4	−26.4	−11.3	−4.9	−36	−4.9
Population estimate	3,400,000	2,350,000	380,000	2,300,000	2,300,000	130,000	5,050,000	265,000	785,000

Species	Long-term trend (%)	10-year trend (%)	Survival change (%)	Population estimate
Blue tit	21	−4	12.6	3,400,000
Great tit	79	−7	2.1	2,350,000
Long-tailed tit	102	0	17.9	380,000
Willow warbler	−43	−3	−26.4	2,300,000
Sedge warbler	−34	−24	−13.6	240,000
Reed warbler	117	−4	−11.3	130,000
Chaffinch	0	−27	−4.9	5,050,000
Bullfinch	−38	21	−36	265,000
Greenfinch	−64	−68	−4.9	785,000

(II) Reduce visual clutter

6. Avoid heavy gridlines

Species	Long-term trend (%)	10-year trend (%)	Survival change (%)	Population estimate
Chaffinch	0	−27	−4.9	5,050,000
Bullfinch	−38	21	−36.0	265,000
Greenfinch	−64	−68	−4.9	785,000

Species	Long-term trend (%)	10-year trend (%)	Survival change (%)	Population estimate
Chaffinch	0	−27	−4.9	5,050,000
Bullfinch	−38	21	−36.0	265,000
Greenfinch	−64	−68	−4.9	785,000

7. Remove unit repetition

Species	Long-term trend	10-year trend	Survival change	Productivity change
Willow warbler	−43%	−3%	−26.4%	12.5%
Sedge warbler	−34%	−24%	−13.6%	41.0%
Reed warbler	117%	−4%	−11.3%	−18.9%
Garden warbler	−11%	−12%	−7.9%	12.5%

Species	Long-term trend (%)	10-year trend (%)	Survival change (%)	Productivity change (%)
Willow warbler	−43	−3	−26.4	12.5
Sedge warbler	−34	−24	−13.6	41.0
Reed warbler	117	−4	−11.3	−18.9
Garden warbler	−11	−12	−7.9	12.5

8. Group similar data

Conservation status	Species	10-year trend (%)	Population estimate
Least concern	Blue tit	−4	3,400,000
Least concern	Great tit	−7	2,350,000
High concern	Willow tit	−33	2,750
High concern	Marsh tit	−24	28,500
Moderate concern	Willow warbler	−3	2,300,000
High concern	Wood warbler	−22	6,500
Least concern	Garden warbler	−12	145,000
Least concern	Chaffinch	−27	5,050,00
Moderate concern	Bullfinch	21	265,000
Least concern	Greenfinch	−68	785,000

Conservation status	Species	10-year trend (%)	Population estimate
High concern	Marsh tit	−24	28,500
	Wood warbler	−22	6,500
	Willow tit	−33	2,750
Moderate concern	Willow warbler	−3	2,300,000
	Bullfinch	21	265,000
Least concern	Blue tit	−4	3,400,000
	Great tit	−7	2,350,000
	Chaffinch	−27	5,050,00
	Greenfinch	−68	785,000
	Garden warbler	−12	145,000

(III) Increase readability

9. Ensure that heads stand out from the body

Species	Long-term trend (%)	10-year trend (%)	Survival change (%)
Willow warbler	−43	−3	−26.4
Sedge warbler	−34	−24	−13.6
Reed warbler	117	−4	−11.3
Garden warbler	−11	−12	−7.9

**Species**	**Long-term trend (%)**	**10-year trend (%)**	**Survival change (%)**
Willow warbler	−43	−3	−26.4
Sedge warbler	−34	−24	−13.6
Reed warbler	117	−4	−11.3
Garden warbler	−11	−12	−7.9

10. Highlight outliers

Species	10-year trend (%)	Population estimate
Cetti’s warbler	101	3,450
Garden warbler	−12	145,000
Grasshopper warbler	−27	12,000
Reed warbler	−4	130,000
Sedge warbler	−24	240,000
Willow warbler	−3	2,300,000
Wood warbler	−22	6,500

Species	10-year trend (%)	Population estimate
**Cetti’s warbler**	**101**	3,450
Garden warbler	−12	145,000
Grasshopper warbler	−27	12,000
Reed warbler	−4	130,000
Sedge warbler	−24	240,000
**Willow warbler**	−3	**2,300,000**
Wood warbler	−22	6,500

11. Highlight statistical significance

Species	Population change (%)		
	1-yr	10-yr	23-yr
Canada Goose	19	−9	81
Egyptian Goose	−8	60	
Greylag Goose	−1	14	169

Species	Population change (%)		
	1-yr	10-yr	23-yr
Canada Goose	19	−9	***81**
Egyptian Goose	−8	***60**	
Greylag Goose	−1	14	***169**

Note: Trends with statistically significant changes are marked in bold with an asterisk

12. Use active, concise titles

Table 1. UK Population trends among doves and pigeons.

Species	Long-term trend (%)	10-year trend (%)	Population estimate
Collared dove	266	−22	810,000
Feral pigeon	0	−14	465,000
Stock dove	127	39	320,000
Turtle dove	−98	−82	3,600
Woodpigeon	121	−2	5,510,000

Table 1. All UK dove and pigeon populations decreased between 2008–2018 except stock doves.

Species	Long-term trend (%)	10-year trend (%)	Population estimate
Collared dove	266	−22	810,000
Feral pigeon	0	−14	465,000
Stock dove	127	39	320,000
Turtle dove	−98	−82	3,600
Woodpigeon	121	−2	5,510,000

13. Orient tables horizontally

Species	10-year trend (%)	Survival change (%)	Productivity change (%)	Population estimate
Barn owl	−31	−9.4	6.8	4,000
Little owl	−46	−9.0	42.6	3,600
Tawny owl	−8	5.6	9.6	50,000

Species	10-year trend (%)	Survival change (%)	Productivity change (%)	Population estimate
Barn owl	−31	−9.4	6.8	4,000
Little owl	−46	−9.0	42.6	3,600
Tawny owl	−8	5.6	9.6	50,000
