# Supplementary material for: Design of tables for the presentation and communication of data in ecological and evolutionary biology
Source: Ecol Evol. 2023 Jul 14;13(7):e10062. doi: 10.1002/ece3.10062 (PMC10346464; doi:10.1002/ece3.10062)
Supplement: Supplementary file 3 — Appendix S3 [file ECE3-13-e10062-s005.pdf]

Supplementary Information 3

Summary of reviewed tables

Journal	Volume	Issue	No. of Papers			No. of Tables			
			Include tables	Do not include tables	Total	Statistical	Text	Data	Total
American Journal of Botany	109	5	7	4	11	5	2	14	21
American Naturalist	199	3	7	4	22	5	7	1	13
Annals of the Missouri Botanical Garden	107	1	3	7	10	1	2	5	8
Annual Review of Ecology, Evolution, and Systematics	42	NA	8	21	29	1	7	3	11
Biodiversity and Conservation	31	7	8	5	13	5	2	16	23
Biological Conservation	267	March	16	4	20	13	11	20	44
Biological Invasions	24	7	18	5	23	12	4	23	39
Biological Journal of the Linnean Society	136	3	5	4	9	7	3	3	13
Biotropica	54	4	18	7	25	14	4	27	45
BMC Biology	20	July	8	10	18	2	2	8	12
BMC Ecology and Evolution	22	June	9	7	16	14	2	10	26
Botanical Journal of the Linnean Society	199	3	6	1	7	1	6	9	16
Conservation Biology	36	1	22	6	28	5	28	10	43
Conservation Letters	15	1	6	4	10	0	9	1	10
Diversity and Distributions	28	4	15	4	19	11	5	9	25
Ecography	2022	3	7	6	13	9	1	5	15

Journal	Volume	Issue	No. of Papers			No. of Tables			
			Include tables	Do not include tables	Total	Statistical	Text	Data	Total
Ecological Monographs	92	1	12	1	13	10	8	10	28
Ecology	103	2	10	7	17	8	3	2	13
Ecology and Evolution	12	6	56	17	73	40	21	68	128
Ecology Letters	25	1	10	9	19	6	5	3	14
Ecosphere	13	4	17	24	41	22	7	18	47
Frontiers in Ecology and the Environment	20	1	2	5	7	0	2	0	2
Functional Ecology	36	3	16	6	22	25	4	11	40
Global Ecology and Biogeography	31	3	10	4	14	12	3	3	18
International Journal of Plant Sciences	183	5	4	2	6	0	0	4	4
Journal of Animal Ecology	91	3	11	3	14	13	1	5	19
Journal of Biogeography	49	4	8	7	15	6	1	4	11
Journal of Ecology	110	2	6	9	15	7	2	3	12
Journal of Tropical Ecology	38	4	6	1	7	16	2	7	25
Journal of Zoology	317	3	7	0	7	19	3	1	23
Methods in Ecology and Evolution	13	2	15	8	23	16	9	10	35
Nature Ecology & Evolution	6	2	4	5	9	1	2	1	4
NeoBiota	NA	74	8	2	10	7	5	7	19
Neotropical Biodiversity	7	1	41	21	62	22	13	66	101
Oecologia	198	4	14	6	20	29	1	6	36
Oikos	2022	4	12	3	15	19	5	6	30
Perspectives in Plant Ecology, Evolution and Systematics	55	6	5	0	5	4	2	5	11

Journal	Volume	Issue	No. of Papers			No. of Tables			
			Include tables	Do not include tables	Total	Statistical	Text	Data	Total
Plant Biology	24	4	14	5	19	0	9	19	28
Plant Ecology and Diversity	15	1-2	5	2	7	4	1	4	9
Plant Systematics and Evolution	308	4	7	1	8	4	4	6	14
Taxon	71	3	5	37	42	1	4	6	11
Trends in Ecology & Evolution	37	3	4	5	9	0	4	1	5
Zoological Journal of the Linnean Society	195	3	6	2	8	2	3	8	13
