# Supplementary material for: Design of tables for the presentation and communication of data in ecological and evolutionary biology
Source: Ecol Evol. 2023 Jul 14;13(7):e10062. doi: 10.1002/ece3.10062 (PMC10346464; doi:10.1002/ece3.10062)
Supplement: Supplementary file 4 — Appendix S4 [file ECE3-13-e10062-s004.pdf]

# Supplementary Information 4
# Detailed tabulation of results

In total, we reviewed 1068 tables across 43 journals. See Supplementary Information 1 for details of the guidelines, and 3 for the journals and tables we reviewed.

## I. Aid comparisons

*1. Left-align text*

| Category | Data | Stats | Text | Total |
|---|---|---|---|---|
| **Yes** | 398 | 394 | 203 | 995 |
| **Yes and No** | 29 | 1 | 16 | 46 |
| **No** | 4 | 1 | 0 | 5 |
| **NA** | 17 | 5 | 0 | 22 |

*2. Left-align text heads*

| Category | Data | Stats | Text | Total |
|---|---|---|---|---|
| **Yes** | 329 | 281 | 158 | 768 |
| **Yes and No** | 34 | 16 | 43 | 93 |
| **No** | 18 | 10 | 13 | 41 |
| **NA** | 67 | 94 | 5 | 166 |

*3. Right-align numbers*

| Category | Data | Stats | Text | Total |
|---|---|---|---|---|
| **Yes** | 55 | 53 | 2 | 256 |
| **Yes and No** | 40 | 44 | 0 | 84 |
| **Within-column variation** | 41 | 13 | 0 | 54 |
| **No** | 236 | 209 | 27 | 472 |
| **NA** | 15 | 2 | 185 | 202 |

*4. Right-align number headers*

| Category | Data | Stats | Text | Total |
|---|---:|---:|---:|---:|
| **Yes** | 3 | 2 | 0 | 5 |
| **No** | 430 | 397 | 33 | 860 |
| **NA** | 15 | 2 | 186 | 203 |

*5. Use the same level of precision*

| Category | Data | Stats | Text | Total |
|---|---:|---:|---:|---:|
| **Yes** | 303 | 242 | 22 | 567 |
| **Yes with exception** | 26 | 37 | 2 | 65 |
| **No** | 103 | 120 | 7 | 230 |
| **NA** | 16 | 2 | 188 | 206 |

*6. Include visualizations if suitable*

| Category | Data | Stats | Text | Total |
|---|---:|---:|---:|---:|
| **Contains graph/chart** | 1 | 0 | 1 | 2 |
| **Contains images** | 0 | 0 | 4 | 4 |
| **Heatmap** | 2 | 1 | 0 | 3 |
| **Contains symbols** | 1 | 0 | 0 | 1 |
| **Cells shaded** | 3 | 5 | 4 | 12 |
| **No** | 441 | 395 | 210 | 1046 |

## II. Reduce visual clutter

*1. Avoid heavy grid lines*

| Category | Data | Stats | Text | Total |
|---|---|---|---|---|
| **Yes** | 273 | 221 | 128 | 622 |
| **Cell shading separates rows/groups** | 161 | 161 | 72 | 394 |
| **No (use of gridlines)** | 14 | 19 | 19 | 52 |

*2. Remove unit repetition within cells*

| Category | Data | Stats | Text | Total |
|---|---|---|---|---|
| **Yes** | 213 | 67 | 3 | 283 |
| **Yes and No** | 4 | 1 | 0 | 5 |
| **No** | 40 | 8 | 6 | 54 |
| **NA** | 191 | 325 | 210 | 726 |

*3. Group similar data*

| Category | Data | Stats | Text | Total |
|---|---|---|---|---|
| **Yes** | 202 | 224 | 99 | 525 |
| **Yes and No** | 3 | 0 | 1 | 4 |
| **No** | 44 | 11 | 33 | 88 |
| **NA** | 199 | 166 | 86 | 451 |

## III. Increase readability

*1. Ensure that headers stand out from the body*

| Category | Data | Stats | Text | Total |
|---|---|---|---|---|
| **Yes** | 440 | 396 | 211 | 1047 |
| **Inconsistent yes** | 3 | 1 | 1 | 5 |
| **Yes and No** | 2 | 2 | 0 | 4 |
| **No** | 3 | 2 | 1 | 6 |
| **NA** | 0 | 0 | 6 | 6 |

*3. Highlight statistical significance*

| Category | Data | Stats | Text | Total |
|---|---|---|---|---|
| **Yes** | 48 | 176 | 1 | 225 |
| **Yes and No** | 0 | 3 | 0 | 3 |
| **No** | 15 | 81 | 0 | 96 |
| **NA** | 385 | 141 | 218 | 744 |

*5. Orient tables horizontally*

| Category | Data | Stats | Text | Total |
|---|---|---|---|---|
| **Yes** | 401 | 375 | 197 | 973 |
| **Yes and No** | 1 | 0 | 0 | 1 |
| **No** | 46 | 26 | 22 | 94 |
