# Supplementary material for: Design of tables for the presentation and communication of data in ecological and evolutionary biology
Source: Ecol Evol. 2023 Jul 14;13(7):e10062. doi: 10.1002/ece3.10062 (PMC10346464; doi:10.1002/ece3.10062)
Supplement: Supplementary file 5 — Appendix S5 [file ECE3-13-e10062-s002.pdf]

Supplementary Information 5

Full summary on journal guidelines on formatting tables

American Journal of Botany

Tables—Include in manuscript file and place immediately after the Literature Cited section.

Tables need to be formatted using the Table feature in Word or in a spreadsheet such as Excel.

Number tables with Arabic numerals followed by a period. Capitalize first word of title; all others, except proper nouns, are lowercase; spell out names of genera and abbreviations on first mention; place period at end. Include study organism (species or group) and geographic location in each caption when appropriate. Place explanatory notes and define all abbreviations below the table after the heading “Note:” or “Notes:”. Place footnotes after the Notes.

Every column must have an appropriately placed heading, with appropriate subheadings. In the body of the table, capitalize the first word of each entry (and proper nouns); do not use vertical lines between columns; indicate footnotes by lowercase superscript letters.

Annals of the Missouri Botanical Garden

- Create tables using the Microsoft Word “Insert Table” feature.
- Tables are neat, double-spaced, and clearly presented.
- Captions are typed double-spaced as paragraphs at the tops of the tables.

- Each table starts on a separate page.

Annual Review of Ecology, Evolution, and Systematics

Only material requiring several columns and several entries should be submitted in tabular form (incorporate other material into the text). Tables should fit within an Annual Reviews page width (6.33 in; approximately 15 cm). Submit editable electronic files for all tables.

All tables will be formatted according to house style. Please adhere to the following guidelines when preparing your tables.

BODY OF TABLE Align entries under the appropriate heading or subheading. Make sure your table clearly indicates the vertical alignment of headings and data. Type longer entries in block style, leaving extra space between entries. Align numbers on the decimal; if numerical data are mixed, center entries in the column. Write out repeated entries or merge cells; do not use ditto marks. Use ND (no data or not determined), NA (not applicable or not available), and NT (not tested) as needed; do not use a dash or leave a cell blank.

FOOTNOTES Footnotes should be at the bottom of the table; label each with a superscript lowercase letter (a, b, c, etc.) keyed to the title, heading, or entry on the table. Begin the lettering anew for each table. If a footnote applies to more than one table, key it to the title of subsequent tables. Include references in the body of the table rather than as footnotes.

ACCEPTABLE FILE TYPES Table files must be compatible with Microsoft Word [.doc(x) or .rtf] or Excel [.xls(x)]. Mathematically complex tables may be submitted in LaTeX.

The illustration on the previous page is a guide for laying out the title, columns, rows, and footnotes for a table.

ARTICLE COMPONENTS: TABLES

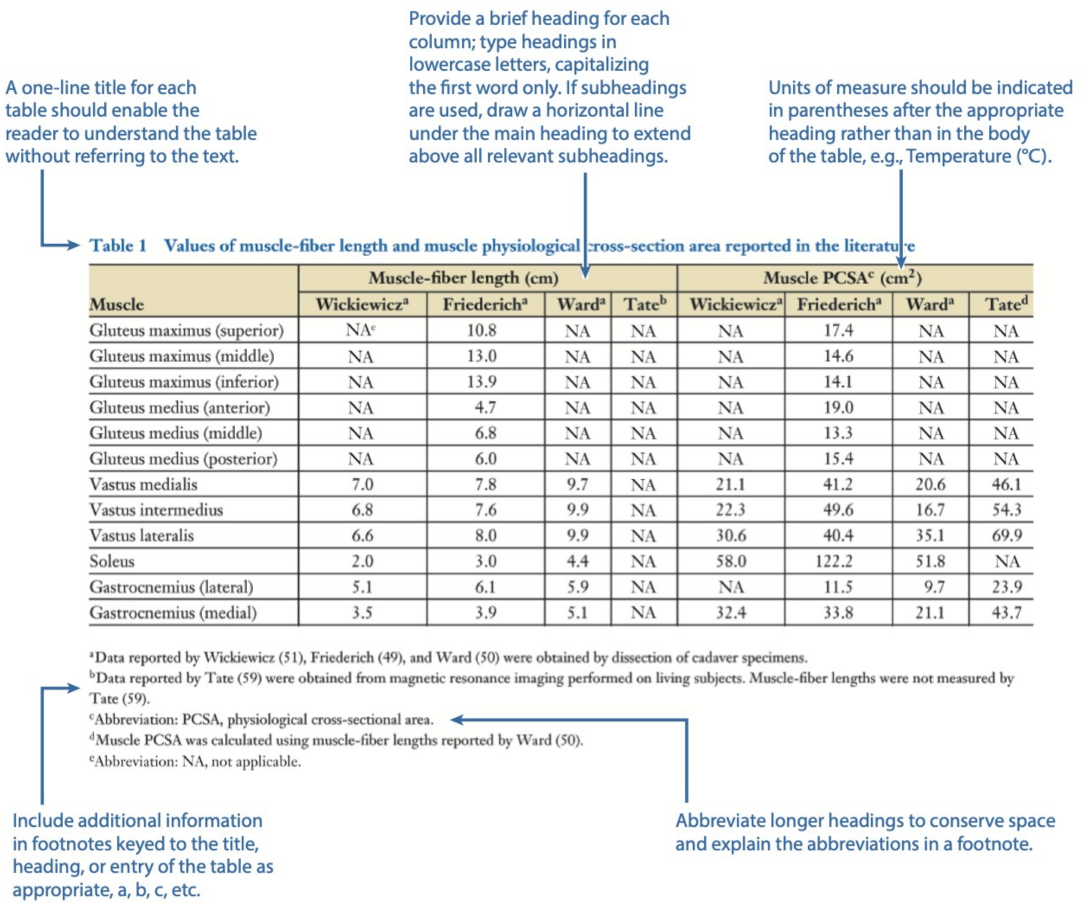

Table 1 Values of muscle-fiber length and muscle physiological cross-section area reported in the literature

	Muscle-fiber length (cm)				Muscle PCSA[c] (cm^2)			
Muscle	Wickiewicz[a]	Friederich[a]	Ward[a]	Tate[b]	Wickiewicz[a]	Friederich[a]	Ward[a]	Tate[d]
Gluteus maximus (superior)	NA[e]	10.8	NA	NA	NA	17.4	NA	NA
Gluteus maximus (middle)	NA	13.0	NA	NA	NA	14.6	NA	NA
Gluteus maximus (inferior)	NA	13.9	NA	NA	NA	14.1	NA	NA
Gluteus medius (anterior)	NA	4.7	NA	NA	NA	19.0	NA	NA
Gluteus medius (middle)	NA	6.8	NA	NA	NA	13.3	NA	NA
Gluteus medius (posterior)	NA	6.0	NA	NA	NA	15.4	NA	NA
Vastus medialis	7.0	7.8	9.7	NA	21.1	41.2	20.6	46.1
Vastus intermedius	6.8	7.6	9.9	NA	22.3	49.6	16.7	54.3
Vastus lateralis	6.6	8.0	9.9	NA	30.6	40.4	35.1	69.9
Soleus	2.0	3.0	4.4	NA	58.0	122.2	51.8	NA
Gastrocnemius (lateral)	5.1	6.1	5.9	NA	NA	11.5	9.7	23.9
Gastrocnemius (medial)	3.5	3.9	5.1	NA	32.4	33.8	21.1	43.7

[a]Data reported by Wickiewicz (51), Friederich (49), and Ward (50) were obtained by dissection of cadaver specimens.
[b]Data reported by Tate (59) were obtained from magnetic resonance imaging performed on living subjects. Muscle-fiber lengths were not measured by Tate (59).
[c]Abbreviation: PCSA, physiological cross-sectional area.
[d]Muscle PCSA was calculated using muscle-fiber lengths reported by Ward (50).
[e]Abbreviation: NA, not applicable.

Biodiversity and Conservation

- All tables are to be numbered using Arabic numerals.
- Tables should always be cited in text in consecutive numerical order.
- For each table, please supply a table caption (title) explaining the components of the table.
- Identify any previously published material by giving the original source in the form of a reference at the end of the table caption.
- Footnotes to tables should be indicated by superscript lower-case letters (or asterisks for significance values and other statistical data) and included beneath the table body.

Biological Conservation

Please submit tables as editable text and not as images. Tables can be placed either next to the relevant text in the article, or on separate page(s) at the end. Number tables consecutively in accordance with their appearance in the text and place any table notes below the table body. Be sparing in the use of tables and ensure that the data presented in them do not duplicate results described elsewhere in the article. Please avoid using vertical rules and shading in table cells.

Biological Invasions

- All tables are to be numbered using Arabic numerals.
- Tables should always be cited in text in consecutive numerical order.
- For each table, please supply a table caption (title) explaining the components of the table.
- Identify any previously published material by giving the original source in the form of a reference at the end of the table caption.
- Footnotes to tables should be indicated by superscript lower-case letters (or asterisks for significance values and other statistical data) and included beneath the table body.

Biological Journal of the Linnean Society

Keep these as simple as possible, with few horizontal and, preferably, no vertical rules. When assembling complex tables and data matrices, bear the dimensions of the printed page (225 x 168 mm) in mind; reducing typesize to accommodate a multiplicity of columns will affect legibility.

Biotropica

While Biotropica does have word limits that differ by manuscript category, there are not have strict limits on the number of tables and/or figures. However, printed manuscripts rarely exceed 32 pages in length, and we encourage authors to submit only necessary tables and figures. Additional

information, figures, and tables should appear in the Supporting Information."

Each table must start on a separate page

Number tables with Arabic numerals followed by a period. Capitalize ‘Table’ (e.g., Table 1, Table 2, etc.).

Indicate footnotes by lowercase superscript letters

Do not use vertical lines in tables.

BMC Biology

When preparing tables, please follow the formatting instructions below.

- Tables should be numbered and cited in the text in sequence using Arabic numerals (i.e. Table 1, Table 2 etc.).
- Tables less than one A4 or Letter page in length can be placed in the appropriate location within the manuscript.
- Tables larger than one A4 or Letter page in length can be placed at the end of the document text file. Please cite and indicate where the table should appear at the relevant location in the text file so that the table can be added in the correct place during production.
- Larger datasets, or tables too wide for A4 or Letter landscape page can be uploaded as additional files. Please see [below] for more information.
- Tabular data provided as additional files can be uploaded as an Excel spreadsheet (.xls) or comma separated values (.csv). Please use the standard file extensions.
- Table titles (max 15 words) should be included above the table, and legends (max 300 words) should be included underneath the table.
- Tables should not be embedded as figures or spreadsheet files, but should be formatted using ‘Table object’ function in your word processing program.

- Color and shading may not be used. Parts of the table can be highlighted using superscript, numbering, lettering, symbols or bold text, the meaning of which should be explained in a table legend.
- Commas should not be used to indicate numerical values.

If you have any questions or are experiencing a problem with tables, please contact the customer service team at info@biomedcentral.com.

BMC Ecology and Evolution

When preparing tables, please follow the formatting instructions below.

- Tables should be numbered and cited in the text in sequence using Arabic numerals (i.e. Table 1, Table 2 etc.).
- Tables less than one A4 or Letter page in length can be placed in the appropriate location within the manuscript.
- Tables larger than one A4 or Letter page in length can be placed at the end of the document text file. Please cite and indicate where the table should appear at the relevant location in the text file so that the table can be added in the correct place during production.
- Larger datasets, or tables too wide for A4 or Letter landscape page can be uploaded as additional files. Please see [below] for more information.
- Tabular data provided as additional files can be uploaded as an Excel spreadsheet (.xls) or comma separated values (.csv). Please use the standard file extensions.
- Table titles (max 15 words) should be included above the table, and legends (max 300 words) should be included underneath the table.
- Tables should not be embedded as figures or spreadsheet files, but should be formatted using ‘Table object’ function in your word processing program.

- Color and shading may not be used. Parts of the table can be highlighted using superscript, numbering, lettering, symbols or bold text, the meaning of which should be explained in a table legend.
- Commas should not be used to indicate numerical values.

If you have any questions or are experiencing a problem with tables, please contact the customer service team at info@biomedcentral.com.

Botanical Journal of the Linnean Society

Keep these as simple as possible, with few horizontal and, preferably, no vertical rules. When assembling complex tables and data matrices, bear the dimensions of the printed page (225 x 168 mm) in mind; reducing typesize to accommodate a multiplicity of columns will affect legibility.

Conservation Biology

A reader should be able to interpret tables and figures without referring to the text and having read only the abstract. The ratio of supporting elements (i.e., tables and figures) to number of words is 1 to 1000. For example, a Contributed Paper of 8000 words should have no more than 8 supporting elements. If a table or figure has only a few data points, incorporate the data into the text.

Text boxes are not allowed.

Tables must not duplicate material in the text or figures and must be editable in Word. Table legends should be one sentence. Additional explanations should be placed in footnotes. Tables should not contain color, gray-scale shading, other graphical elements, or have numbered or lettered parts.

Figure legends should describe the relationship shown (e.g., Coefficients of habitat selection relative to resource selection functions for caribou individuals.). Report results in the Results section, not figure legends.

Before publication, you will be required to supply figures in tif, eps, or pdf format.

Author portrayals of borders or other jurisdictional boundaries on maps in published articles do not imply support of those representations by the journal or SCB.

For guidance on best practices in graphic design, refer to the following link used with permission from *Oryx* - The International Journal of Conservation and Fauna & Flora International: http://scalar.usc.edu/works/graphics-for-conservation/index.

Conservation Letters

NA

Diversity and Distributions

Tables should be self-contained and complement, not duplicate, information contained in the text. They should be supplied as editable files, not pasted as images. Legends should be concise but comprehensive – the table, legend, and footnotes must be understandable without reference to the text, giving the study organism and study location and 'n' values where applicable. Column headings should be brief, with units of measurement in parentheses. All abbreviations must be defined in footnotes.

Ecography

At initial submission, figures, photographs and drawings can be provided within the manuscript or as separate files. For revised manuscripts, illustrations should be uploaded as separate files. The file size limit is 50 MB. Larger files (for example higher resolution) can be provided after acceptance.

All figures and tables should be numbered and referred to in the text by their number. Figure and table captions should be provided within the manuscript, and should be brief and informative, and include any relevant copyright information if taken from a published source.

[…]
Tables and legends of illustrations should be written double-spaced on separate sheets. Do not incorporate the legend in the figure itself. Tables and illustration legends should be comprehensible without reference to the text. Do not use italic lettering.

Ecological Monographs

The following guidelines ensure tables are formatted correctly.

General Information

- Tables must be placed in the Main Document, appearing in their own section after the References section.
- Tables should be numbered in the order of their discussion in the text. Each table should begin on a new page.
- Tables must fit on a page in portrait orientation, widthwise.
- Manuscripts must have “true” tables in an editable format, created by using the “Insert Table” function, rather than using tabs or spaces and should not appear as “pictures” imported from another program.
- In Microsoft Word you should select “Insert”, “Table”, then specify the number of rows and columns and fill in the individual cells.

Table Components and Format

- Table captions should consist of one sentence that will appear above the body of the table. Rather than simply repeating the labels

on columns and rows of the table, this caption should reveal the point of grouping certain data in the table. Statistical values and other pertinent details should be provided as notes/footnotes rather than appearing in the table's caption.

- Additional details should be placed in footnotes and/or a Notes section presented at the bottom of the table.
- Abbreviations used in the body of the table should be identified in an Abbreviations section at the bottom of the table, in the order in which they appear in the table.
- Tables cannot contain colors, shading, or graphics. Use type style (italics, boldface, etc.) to convey meaning and identify the meaning in a Notes section at the bottom of the table (e.g., "Values in boldface indicate significant differences.")
- Tables must be presented in a grid format, with the same number of columns in each row and the same number of rows in each column, below the column headings. Columns should read fully down, and rows should read fully across.
- All columns and rows must be labeled.
- All column headings should identify what is found in the column
- Subparts within the body of a table can be distinguished using (a) (b) etc. labeling and/or nested labeling within the left-most column.
- Table captions, notes, and footnotes must be double-spaced in 12-point Times New Roman font.
- The font size within the body of a table can be reduced to a minimum of 10-point Times New Roman if needed for the table to fit in portrait orientation.

- Table pages should be presented in portrait orientation.
- Tables must not extend to multiple pages in the horizontal direction but may extend in the vertical direction.

Ecology

The following guidelines ensure tables are formatted correctly.

General Information

- Tables must be placed in the Main Document, appearing in their own section after the References section.
- Tables should be numbered in the order of their discussion in the text. Each table should begin on a new page.
- Tables must fit on a page in portrait orientation, widthwise.
- Manuscripts must have “true” tables in an editable format, created by using the “Insert Table” function, rather than using tabs or spaces and should not appear as “pictures” imported from another program.
- In Microsoft Word you should select “Insert”, “Table”, then specify the number of rows and columns and fill in the individual cells.

Table Components and Format

- Table captions should consist of one sentence that will appear above the body of the table. Rather than simply repeating the labels on columns and rows of the table, this caption should reveal the point of grouping certain data in the table. Statistical values and other pertinent details should be provided as notes/footnotes rather than appearing in the table’s caption.
- Additional details should be placed in footnotes and/or a Notes section presented at the bottom of the table.

- Abbreviations used in the body of the table should be identified in an Abbreviations section at the bottom of the table, in the order in which they appear in the table.

- Tables cannot contain colors, shading, or graphics. Use type style (italics, boldface, etc.) to convey meaning and identify the meaning in a Notes section at the bottom of the table (e.g., “Values in boldface indicate significant differences.”)

- Tables must be presented in a grid format, with the same number of columns in each row and the same number of rows in each column, below the column headings. Columns should read fully down, and rows should read fully across.

- All columns and rows must be labeled.

- All column headings should identify what is found in the column

- Subparts within the body of a table can be distinguished using (a) (b) etc. labeling and/or nested labeling within the left-most column.

- Table captions, notes, and footnotes must be double-spaced in 12-point Times New Roman font.

- The font size within the body of a table can be reduced to a minimum of 10-point Times New Roman if needed for the table to fit in portrait orientation.

- Table pages should be presented in portrait orientation.

- Tables must not extend to multiple pages in the horizontal direction but may extend in the vertical direction.

Ecology and Evolution

All tables must be cited in the text in the order that they should appear.

Ecology Letters

Tables should be submitted as MS Excel or MS Word documents (or equivalent). Tables should be cited consecutively in the text, numbered with Arabic numerals (Table 1, Table 2, etc.) and should be grouped together at the end of the paper or in a separate file(s). Each table should be titled and typed double-spaced on a separate sheet. Captions should not exceed 150 words per table (footnote included). Units must be clearly indicated for each of the entries in the table. Footnotes to tables should be identified by the symbols * † ‡ § ¶ (in that order) and placed at the bottom of the table. No vertical rules should be used.

Ecosphere

The following guidelines ensure tables are formatted correctly.

General Information

- Tables must be placed in the Main Document, appearing in their own section after the References section.
- Tables should be numbered in the order of their discussion in the text. Each table should begin on a new page.
- Tables must fit on a page in portrait orientation, widthwise.
- Manuscripts must have “true” tables in an editable format, created by using the “Insert Table” function, rather than using tabs or spaces and should not appear as “pictures” imported from another program.
- In Microsoft Word you should select “Insert”, “Table”, then specify the number of rows and columns and fill in the individual cells.

Table Components and Format

- Table captions should consist of one sentence that will appear above the body of the table. Rather than simply repeating the labels on columns and rows of the table, this caption should reveal the point of grouping certain data in the table. Statistical values and other pertinent details should be provided as notes/footnotes rather than appearing in the table's caption.

- Additional details should be placed in footnotes and/or a Notes section presented at the bottom of the table.

- Abbreviations used in the body of the table should be identified in an Abbreviations section at the bottom of the table, in the order in which they appear in the table.

- Tables cannot contain colors, shading, or graphics. Use type style (italics, boldface, etc.) to convey meaning and identify the meaning in a Notes section at the bottom of the table (e.g., "Values in boldface indicate significant differences.")

- Tables must be presented in a grid format, with the same number of columns in each row and the same number of rows in each column, below the column headings. Columns should read fully down, and rows should read fully across.

- All columns and rows must be labeled.

- All column headings should identify what is found in the column

- Subparts within the body of a table can be distinguished using (a) (b) etc. labeling and/or nested labeling within the left-most column.

- Table captions, notes, and footnotes must be double-spaced in 12-point Times New Roman font.

- The font size within the body of a table can be reduced to a minimum of 10-point Times New Roman if needed for the table to fit in portrait orientation.

- Table pages should be presented in portrait orientation.

- Tables must not extend to multiple pages in the horizontal direction but may extend in the vertical direction.

Frontiers in Ecology and the Environment

- Tables of reasonable size and sidebars (panels) containing extra information are also welcomed; very large tables may have to be displayed as Web-only material on the electronic version of the journal
- Do not place tables within the manuscript text; these should be placed after the References and figure captions
- Try to limit tables to 200 words and five columns; if you have more information than this, please consider, in order of preference, (1) trimming down the information, (2) dividing it into multiple tables, or (3) contacting editorial staff for guidance on Web-only material (see also below)

Functional Ecology

- Figures and Tables can be embedded within the text where referenced to facilitate reviewing.

  […]

- **Results** - State the results and draw attention in the text to important details shown in tables and figures.

Global Ecology and Biogeography

Tables should be self-contained and complement, not duplicate, information contained in the text. They should be supplied as editable

files, not pasted as images. Legends should be concise but comprehensive – the table, legend, and footnotes must be understandable without reference to the text, giving the study organism and study location and 'n' values where applicable. Column headings should be brief, with units of measurement in parentheses. All abbreviations must be defined in footnotes.

International Journal of Plant Sciences

Creating

Use your programs table-creation tool for all tables. Do not use tabs and spaces. Do not break contents of a cell across multiple cells. Cell entries should clearly belong to a specific row and column.

Formatting

Tables should be prepared using a roman font; italic and bold should not be used for emphasis. Except for basic horizontal lines (see "Lines"), tables should be free of lines, boxes, arrows, or other devices unless they indicate the structure of the data.

Numbering

Tables are numbered using arabic numerals, and they are cited in numerical order in the text. Appendix tables are generally numbered as follows: if there is only one appendix in the article, tables in the appendix are numbered A1, A2, A3, etc. If there are more appendixes, tables are numbered to correspond to the appendix in which they occur; that is, Appendix B would contain tables B1, B2, etc.; Appendix C would contain tables C1, C2, and so on.

Table titles

Titles should identify the table as briefly as possible. In an article that contains more than one table, each title should be unique. Titles should not contain explanatory material; this should be placed in a "note" at the bottom of the table.

Column headings

Headings should identify the column as briefly as possible. Headings should contain any necessary symbols (%, $, etc.) or measurement abbreviations (cm, kg, etc.) that apply to the data in the column below. Any measurement abbreviations should conform to the style of the journal. Headings may have several levels, with horizontal rules separating the levels (see example A below).

Example A

Table 4

SEEMINGLY UNRELATED REGRESSIONS OF PARENTING WITH INTERACTION EFFECTS FOR MARITAL STATUS, CORESIDENCE, AND FIRST BIRTH[a]

	ENGAGEMENT		SPANKING	
	Coefficient	SE	Coefficient	SE
By marital status (full sample):				
Mother's parenting:				
Supportiveness	.43*	.18	.02	.11
Change in supportiveness	.34*	.14	−.23**	.08
Conflict	.27*	.14	.09	.09
Married × supportiveness	.21	.29	−.17	.18
Married × change in supportiveness	−.38	.25	.14	.16
Married × conflict	−.29	.25	.11	.16
Father's parenting:				
Supportiveness	.79**	.22	−.11	.10
Change in supportiveness	.53**	.17	.02	.08
Conflict	−.44*	.17	.07	.08
Married × supportiveness	−.19	.36	.10	.17
Married × change in supportiveness	.06	.31	−.03	.15
Married × conflict	.51	.31	.06	.15
By cohabiting vs. visiting (unmarried sample only):				
Mother's parenting:				
Supportiveness	.71*	.32	−.12	.21
Change in supportiveness	.57*	.27	−.49**	.17
Conflict	.58*	.25	−.07	.16
Cohabiting × supportiveness	−.30	.39	.18	.25
Cohabiting × change in supportiveness	−.26	.31	.36[+]	.20
Cohabiting × conflict	−.45	.30	.20	.19
Father's parenting:				
Supportiveness	1.20**	.40	−.02	.19
Change in supportiveness	.75*	.33	.13	.16
Conflict	−.33	.31	−.05	.15
Cohabiting × supportiveness	−.57	.49	−.11	.23
Cohabiting × change in supportiveness	−.26	.39	−.15	.19
Cohabiting × conflict	−.12	.37	.17	.18
By first birth vs. later birth (unmarried sample only):				
Mother's parenting:				
Supportiveness	.55*	.23	−.02	.14
Change in supportiveness	.45**	.17	−.22*	.11
Conflict	.22	.17	.06	.11
First birth × supportiveness	−.14	.37	.13	.23
First birth × change in supportiveness	−.18	.28	−.01	.18
First birth × conflict	.14	.29	.04	.19
Father's parenting:				
Supportiveness	.76*	.30	−.22	.14
Change in supportiveness	.61**	.23	−.02	.11
Conflict	−.36	.24	.10	.11
First birth × supportiveness	.15	.45	.26	.21
First birth × change in supportiveness	−.16	.35	.08	.16
First birth × conflict	−.14	.35	−.06	.16

NOTE.—Each model includes all other independent variables shown in table 3 (age, race and ethnicity, family background, first birth, education, employment, poverty status, health status, substance problem, children in household, religious attendance, marital status at birth, prenatal care [mother models only], child sex, child temperament, and other parent's engagement or spanking).

[a] Models for mothers and fathers are jointly estimated for engagement and spanking, respectively.

[+] $p < .10$.

* $p < .05$.

** $p < .01$.

If column numbers are mentioned in text, table columns should be numbered accordingly. Numbering should begin with the first column to the right of the stub column.

Lines

Only horizontal lines are allowed; no vertical lines, boxes, or other lines may be used unless they indicate the structure of the data. All tables should have the following three horizontal lines:

- One under the title, above the column headings
- One between the column headings and the body of the table
- One at the bottom of the table.

In addition, tables MAY have the following horizontal lines, as needed:

- To separate levels when there is more than one level of column heading
- To separate a column of numbers that is being added from its total (see example B below)
- To delineate "cut-in" heads in the body of a table (see "cut-in heads" below).

Example B

Table 4

RATES OF DHS SHELTER USE BY SELECTED CHARACTERISTICS

	Either Shelter System* (%)	Family Shelter System (%)	Single Adult Shelter System (%)
History of out-of-home care:			
Yes	22.4	17.0	8.9
No	10.8	9.4	2.5
Type of final exit from ACS:			
Reunification	19.4	14.7	7.6
Independent living	25.6	18.8	10.7
Absconding from care	33.6	22.4	15.6
Preventive services	12.4	11.0	3.0
Race and ethnicity:			
African American (non-Hispanic)	24.3	18.8	8.9
Hispanic	14.1	11.0	5.2
Caucasian (non-Hispanic)	6.0	4.0	3.0
Gender:			
Female	25.1	23.7	4.8
Male	11.1	3.8	9.3
Total	18.7	14.5	6.9

NOTE.—DHS = Department of Homeless Services; ACS = the New York City Administration for Children's Services. All relationships are statistically significant for χ^2 test ($p < .001$).

* "Either shelter system" category reflects the unduplicated sum of the other two columns.

Stub column (the left-most column in the table)

Entries should be as brief as possible. Stub column entries are often layered (see example A). Note the use of colons and indentations to differentiate the various layers. Stub column entries and subentries should

be confined to one column. Data in columns to the right of the stub column should be in the same row as the stub column entry. Stub column entries should not run across into the body of the table. If necessary, the entries are broken and runover lines are indented.

Cut-in heads

Cut-in heads are used when the contents of columns change-that is, when new labels are needed for the data in the columns. This is the only circumstance in which headings should appear in the body of the table. See example C below for the correct use of cut-in heads.

Example C

Table 1

TRENDS IN JOINT CUSTODY, CHILD SUPPORT PAYMENTS, AND VISITATION

	YEAR DIVORCED			
	1981–84	1985–88	1989–92	1993–98
Legal custody status (%):				
Joint custody	20.9	27.4	31.2	34.2
Mother, sole custody	66.3	60.7	57.3	53.5
Father, sole custody	12.8	11.9	11.5	12.2
	SURVEY YEAR			
	1992	1994	1996	1998
Child support payments (real 1997 $):				
Joint custody	2,390	2,526	2,656	2,753
Mother, sole custody	1,956	1,994	1,989	1,990
Father, sole custody	401	826	952	667
Visitation (days with non-resident parent):				
Joint custody	80.4	74.4	70.8	85.8
Mother, sole custody	34.1	34.4	37.2	39.1
Father, sole custody	45.4	48.8	46.4	56.3

NOTE.—1992–98 CPS-CSS. Sample limits to parents ages 15–65.

Rules are used above and below cut-in heads. Note that these rules cover only the columns that change. They do NOT extend into the stub column. As with headings at the top of a table, cut-in heads may have more than one level.

Total rule
A total rule is to be used only when there is a total that is actually the SUM of the numbers in a column. It is not to be used for averages, means, or other numbers that may summarize the data in a column, but that are not totals. See example B. The total rule extends only across columns with totals. It should not extend into other columns that do not have totals. Missing data: Three centered ellipses may be used in blank cells; it is also acceptable to leave these cells empty.

Body of the table
Wherever possible, the body of the table should be free of symbols (%, $, etc.) or measurement abbreviations (cm, kg, etc.). Symbols should appear in the column head when they apply to all values in the column, or in the stub column when they apply to all values in the row. Sometimes they can even be placed at the end of the table title, if they apply to all of the data within the table.

Footnotes to tables
All notes end with a period, even if they are not complete sentences. Table note callouts should be arranged in the body of the table from left to right, top to bottom, as if reading a text. Every callout in the table should have a corresponding footnote. The type of footnote symbols used (numbers, letters, symbols) depends on the style of the journal. See individual journals' "Information for Contributors" for specific instructions. Many tables will have a general explanatory "note" containing useful information about the table (definitions of abbreviations used, etc.). This note is not numbered. It is simply labeled "note" and appears at the bottom of the table before any other specific, numbered notes. The word "note" is singular, even when there are multiple pieces of information in the same note. See example D below.

Example D

Table 5

ESTIMATES OF THE RELATIONSHIP BETWEEN MATERNAL WORK HOURS AND THE PROBABILITY IN SELF-CARE, NONLINEAR SPECIFICATION OF WORK HOURS: LOW-SES MOTHERS

MATERNAL WORK HOURS (per Week)	LOW EDUCATION		LOW INCOME	
	Logit	FE Logit	Logit	FE Logit
At most 10	−.272	−.112	.102	.194
	(.324)	(.597)	(.436)	(.821)
More than 10 and at most 20	.300	.225	.553*	.162
	(.189)	(.335)	(.238)	(.437)
More than 20 and at most 30	.618**	.199	.423*	.086
	(.158)	(.291)	(.215)	(.384)
More than 30 hours	1.358**	1.011**	1.309**	.897**
	(.086)	(.190)	(.104)	(.231)
	[.197]	[.057]	[.178]	[.108]
N	6,059	1,383	4,020	873

SOURCE.—Author's calculations using the National Longitudinal Survey of Youth, 1979 cohort.

NOTE.—AFQT = Armed Forces Qualification Test; FE = fixed effects; SES = socioeconomic status. Huber-White standard errors are reported in parentheses, and they are corrected to account for the intraindividual correlation; marginal effects for statistically significant maternal work hours coefficients reported in brackets; mothers in the low-education subsample had less than or equal to a high school education in the first year their adolescent was observed; mothers in the poor subsample had family incomes below the poverty threshold in either 1985, 1986, or 1987. All models contain controls for the adolescent's age, the mother's highest grade completed, the mother's marital status, the number of adults and the number of children in the mother's household, and the year; the logit models also include controls for the adolescent's sex, African American, Hispanic, the mother's AFQT score, if the mother has traditional values, and the mother's age when she had her first child.

* $p < .05$.

** $p < .01$.

Some tables also have an unnumbered note labeled "sources" (or "source" if only one source is cited). This is used to list the sources that were used in constructing the table. If such a note is used, it should precede all other footnotes, including the "general" note. See example D above. Footnotes indicating standard levels of significance in statistical tables usually use asterisks. These footnotes should follow all other footnotes at the bottom of the table. See example D.

Journal of Animal Ecology

- Figures and tables can be embedded within the text where referenced and should appear with their legends to facilitate reviewing
- […]
- Results - State the results and draw attention in the text to important details shown in tables and figures.

Journal of Biogeography

Tables should be self-contained and complement, not duplicate, information contained in the text. They should be supplied as editable files, not pasted as images. Legends should be concise but comprehensive – the table, legend, and footnotes must be understandable without reference to the text, giving the study organism and study location and 'n' values where applicable. Column headings should be brief, with units of measurement in parentheses. All abbreviations must be defined in footnotes.

Journal of Ecology

- Figures and Tables can be embedded within the text where referenced to facilitate reviewing

Journal of Tropical Ecology

Figure and table legends should be concise but informative, ideally allowing readers to comprehend what the figure/table represents without reference to the main text of the paper. Each table should be provided on a separate page after the References section. The figures should be supplied as separate files in TIF, PDF, or EPS format at approximate final publication size (please see a recent article in the journal for column widths). Artwork should be at the following minimum resolutions: line artwork (black & white), 1200 dpi; combination, i.e. line/tone (greyscale), 800 dpi; black-and-white halftone (greyscale), 300 dpi; and colour halftone, 300 dpi. Colour is only encouraged where its use increases comprehension of the figure. All wording within submitted figures must be Arial 8 pt font. Figure captions should be supplied at the end of the article, not as part of the figure files. Where possible put keys to symbols and lines in legends, not on figures.

Journal of Zoology

Tables must fit the page size (220 x 168 mm) without undue reduction. Oversize tables will not be accepted. Tables are referred to as Table 1,

Table 2, etc., and any sub-sections as (a), (b), etc. Footnotes should be indicated by superscript a, b.

Methods in Ecology and Evolution

Figures, including photographs, should be referred to in the article text as Fig. 1, Figs 2–4. References to tables should not be abbreviated, i.e. Table 1. All lettering and symbols must be clear and easy to read. Legends should provide enough details for the figure or table to be understood without reference to the main text. Information (e.g. keys) that appear in the figure should not be duplicated in the legend.

Figures and Tables should be presented in the manuscript file with their legends and may be either embedded in a relevant position in the main text or placed at the end of the document.

Nature Ecology and Evolution

- Please include tables at the end of your text document.
- Complex tables can be submitted as a separate Excel file.
- Tables that include statistical analysis of data should describe their standards of error analysis and ranges in a table legend.
- Tables that feature chemical structures should be included at the end of the text document and the native ChemDraw file(s) should be supplied separately as .cdx files.

NeoBiota

Tables: Each table should be numbered in sequence using Arabic numerals (i.e. Table 1, 2, 3 etc.). Tables should also have a title that summarizes the whole table, maximum 15 words. Detailed legends may then follow, but should be concise.

Small tables can be embedded within the text, in portrait format (note that tables on a landscape page must be reformatted onto a portrait page or submitted as additional files). These will be typeset and displayed in the final published form of the article. Such tables should be formatted

using the 'Table object' in a word processing program to ensure that columns of data are kept aligned when the file is sent electronically for review. Do not use tabs to format tables or separate text. All columns and rows should be visible, please make sure that borders of each cell display as black lines. Colour and shading should not be used; neither should commas be used to indicate decimal values. Please use a full stop to denote decimal values (i.e., 0.007 cm, 0.7 mm).

Larger datasets can be uploaded separately as Supplementary Files. Tabular data provided as supplementary files can be uploaded as an Excel spreadsheet (.xls), as an OpenOffice spreadsheets (.ods) or comma separated values file (.csv). As with all uploaded files, please use the standard file extensions.

Neotropical Biodiversity

Tables. Tables should present new information rather than duplicating what is in the text. Readers should be able to interpret the table without reference to the text. Please supply editable files.

Oecologia

Tables Each table should be submitted on a separate page, with the title (heading) above the table. Tables should be understandable without reference to the manuscript text. Restrict your use of tables to essential material. All tables must be cited in the manuscript text and numbered consecutively with Arabic numerals. Provide dimensions or units for all numbers. Identify any previously published material by giving the original source in the form of a reference at the end of the table heading. Tables will be printed with horizontal separation lines only (one below the table's header, one below the column headers, and one at the end of the table); no vertical lines will be printed. Use tab stops to align columns and center numbers around decimals when appropriate. Footnotes to tables should be indicated by superscript lower-case letters (or asterisks for significance values and other statistical data). The number of decimals presented

should be sensible and match the precision of the data. Acceptable file formats for tables include Microsoft Word (.doc), Rich Text Format (.rtf) and Excel (.xls).

Oikos

At initial submission, figures, photographs and drawings can be provided within the manuscript or as separate files. For revised manuscripts, illustrations should be uploaded as separate files. The file size limit is 50 MB. Larger files (for example higher resolution) can be provided after acceptance.

All figures and tables should be numbered and referred to in the text by their number. Figure and table captions should be provided within the manuscript, and should be brief and informative, and include any relevant copyright information if taken from a published source.

[…]

Tables and legends of illustrations should be written double-spaced on separate sheets. Do not incorporate the legend in the figure itself. Tables and illustration legends should be comprehensible without reference to the text. Do not use italic lettering.

Be consistent throughout the figure with colours, line weights, and styles. Panels within the figure should be designated with lower case letters in parentheses (e.g. (a), (b), (c)...).

The ScholarOne submission system does not accept individual image file > 50 MB. However, larger files (e.g. high-resolution photographs of plant specimens) can be provided after acceptance. Please contact the managing editor (ecography[at]oikosoffice.lu.se) for instructions.

Colour figures are most welcome and will be published free of charge. However, we urge all authors to create figures that are accessible for all types of colour vision. When creating a figure use the following set of simple rules: 1) use a colour-blind safe palette (e.g. avoid using red and

green together), 2) use high contrast, 3) in fluorescent red-green images, replace red with magenta, 4) check your figure using one of the many free tools that allow you to see how it looks for the colour blind, 5) consider alternative ways that do not rely on colour to visualize your data. For example, you might want to use monochromatic figures, or different shapes, positions and line types instead. You can make use of R-script colour palettes and Python colour blindness palettes. More information about how to make figures that are colour blindness friendly can be found here.

Perspectives in Plan Ecology, Evolution and Systematics

Please submit tables as editable text and not as images. Tables can be placed either next to the relevant text in the article, or on separate page(s) at the end. Number tables consecutively in accordance with their appearance in the text and place any table notes below the table body. Be sparing in the use of tables and ensure that the data presented in them do not duplicate results described elsewhere in the article. Please avoid using vertical rules and shading in table cells.

Plant Biology

Tables must be serially numbered in Arabic numerals and each must carry a brief descriptive heading. Tables reproduced from other publications must state their precise source. Only signs that can be typeset should be used in the tables and legends. Please provide Tables in Word and include them at the end of the manuscript after the References, each on a separate page. Refrain from using both tables and graphs to demonstrate the same results.

Plant Ecology & Diversity

Tables. Tables should present new information rather than duplicating what is in the text. Readers should be able to interpret the table without reference to the text. Please supply editable files.

Plan Systematics and Evolution

- All tables are to be numbered using Arabic numerals.
- Tables should always be cited in text in consecutive numerical order.
- For each table, please supply a table caption (title) explaining the components of the table.
- Identify any previously published material by giving the original source in the form of a reference at the end of the table caption.
- Footnotes to tables should be indicated by superscript lower-case letters (or asterisks for significance values and other statistical data) and included beneath the table body.

PSE encourages online-only publication of extensive tables that support the article but more convenient in electronic form (see Electronic Supplementary Material).

Taxon

Tables are to be prepared using MS Word’s Table function or by using single tabs between columns, but no additional tabs or spaces to adjust entries. Do not split lengthy tables into parts. Tables must have an appropriate heading and must bear consecutive Arabic numerals. They are cited “Table 1”, etc. Long tables including plant materials or accessory materials are designed as an Appendix and run in paragraphs (order alphabetically by taxon name; see recent issue of TAXON for style). Lengthy Tables and Appendices are provided as Supporting Information to the online version of the article and are not published in print. When there are several figures (e.g., several trees of individual and combined datasets), authors should indicate the ones they feel are most important and should be in the printed version, and those which should appear as Supporting Information.

The American Naturalist

Tables can be embedded in the flow of text in the review manuscript. For Production they appear after the Literature Cited and before the figure legends.

- Do not present the same information in both a table and a figure.
- Table titles must be short, concise, and descriptive. All other information should be placed in a table note.
- Table notes should appear after the table.
- Print tables are numbered consecutively in the order in which they appear in the text. All print tables must be referred to in the text.
- There are no vertical or horizontal lines in the body of an *American Naturalist* table. There are no panels. There is no graphical representation of any kind, including colors or shading. There is no fixed spatial arrangement of elements. If a table must have a graphical aspect, then it should be renamed a figure.
- A table has the same column headings throughout. If the column headings change, it is a new table with a new table number and a new table title.
- Sequences should be taxonomic with family headings or alphabetical by scientific name.
- Online edited and typeset tables should follow the same rules.
- In Word, tables must be entered using the table editor.

Creating

Use your programs table-creation tool for all tables. Do not use tabs and spaces. Do not break contents of a cell across multiple cells. Cell entries should clearly belong to a specific row and column.

Formatting

Tables should be prepared using a roman font; italic and bold should not be used for emphasis. Except for basic horizontal lines (see "Lines"), tables should be free of lines, boxes, arrows, or other devices unless they indicate the structure of the data.

Numbering

Tables are numbered using arabic numerals, and they are cited in numerical order in the text. Appendix tables are generally numbered as follows: if there is only one appendix in the article, tables in the appendix are numbered A1, A2, A3, etc. If there are more appendixes, tables are numbered to correspond to the appendix in which they occur; that is, Appendix B would contain tables B1, B2, etc.; Appendix C would contain tables C1, C2, and so on.

Table titles

Titles should identify the table as briefly as possible. In an article that contains more than one table, each title should be unique. Titles should not contain explanatory material; this should be placed in a "note" at the bottom of the table.

Column headings

Headings should identify the column as briefly as possible. Headings should contain any necessary symbols (%, $, etc.) or measurement abbreviations (cm, kg, etc.) that apply to the data in the column below. Any measurement abbreviations should conform to the style of the journal. Headings may have several levels, with horizontal rules separating the levels (see example A below).

Example A

Table 4

SEEMINGLY UNRELATED REGRESSIONS OF PARENTING WITH INTERACTION EFFECTS FOR MARITAL STATUS, CORESIDENCE, AND FIRST BIRTH[a]

	ENGAGEMENT		SPANKING	
	Coefficient	SE	Coefficient	SE
By marital status (full sample):				
Mother's parenting:				
Supportiveness	.43*	.18	.02	.11
Change in supportiveness	.34*	.14	−.23**	.08
Conflict	.27*	.14	.09	.09
Married × supportiveness	.21	.29	−.17	.18
Married × change in supportiveness	−.38	.25	.14	.16
Married × conflict	−.29	.25	.11	.16
Father's parenting:				
Supportiveness	.79**	.22	−.11	.10
Change in supportiveness	.53**	.17	.02	.08
Conflict	−.44*	.17	.07	.08
Married × supportiveness	−.19	.36	.10	.17
Married × change in supportiveness	.06	.31	−.03	.15
Married × conflict	.51	.31	.06	.15
By cohabiting vs. visiting (unmarried sample only):				
Mother's parenting:				
Supportiveness	.71*	.32	−.12	.21
Change in supportiveness	.57*	.27	−.49**	.17
Conflict	.58*	.25	−.07	.16
Cohabiting × supportiveness	−.30	.39	.18	.25
Cohabiting × change in supportiveness	−.26	.31	.36+	.20
Cohabiting × conflict	−.45	.30	.20	.19
Father's parenting:				
Supportiveness	1.20**	.40	−.02	.19
Change in supportiveness	.75*	.33	.13	.16
Conflict	−.33	.31	−.05	.15
Cohabiting × supportiveness	−.57	.49	−.11	.23
Cohabiting × change in supportiveness	−.26	.39	−.15	.19
Cohabiting × conflict	−.12	.37	.17	.18
By first birth vs. later birth (unmarried sample only):				
Mother's parenting:				
Supportiveness	.55*	.23	−.02	.14
Change in supportiveness	.45**	.17	−.22*	.11
Conflict	.22	.17	.06	.11
First birth × supportiveness	−.14	.37	.13	.23
First birth × change in supportiveness	−.18	.28	−.01	.18
First birth × conflict	.14	.29	.04	.19
Father's parenting:				
Supportiveness	.76*	.30	−.22	.14
Change in supportiveness	.61**	.23	−.02	.11
Conflict	−.36	.24	.10	.11
First birth × supportiveness	.15	.45	.26	.21
First birth × change in supportiveness	−.16	.35	.08	.16
First birth × conflict	−.14	.35	−.06	.16

NOTE.—Each model includes all other independent variables shown in table 3 (age, race and ethnicity, family background, first birth, education, employment, poverty status, health status, substance problem, children in household, religious attendance, marital status at birth, prenatal care [mother models only], child sex, child temperament, and other parent's engagement or spanking).

[a] Models for mothers and fathers are jointly estimated for engagement and spanking, respectively.

+ $p < .10$.

* $p < .05$.

** $p < .01$.

If column numbers are mentioned in text, table columns should be

numbered accordingly. Numbering should begin with the first column to the right of the stub column.

Lines

Only horizontal lines are allowed; no vertical lines, boxes, or other lines may be used unless they indicate the structure of the data. All tables should have the following three horizontal lines:

- One under the title, above the column headings
- One between the column headings and the body of the table
- One at the bottom of the table.

In addition, tables MAY have the following horizontal lines, as needed:

- To separate levels when there is more than one level of column heading
- To separate a column of numbers that is being added from its total (see example B below)
- To delineate "cut-in" heads in the body of a table (see "cut-in heads" below).

Example B

Table 4

RATES OF DHS SHELTER USE BY SELECTED CHARACTERISTICS

	Either Shelter System* (%)	Family Shelter System (%)	Single Adult Shelter System (%)
History of out-of-home care:			
Yes	22.4	17.0	8.9
No	10.8	9.4	2.5
Type of final exit from ACS:			
Reunification	19.4	14.7	7.6
Independent living	25.6	18.8	10.7
Absconding from care	33.6	22.4	15.6
Preventive services	12.4	11.0	3.0
Race and ethnicity:			
African American (non-Hispanic)	24.3	18.8	8.9
Hispanic	14.1	11.0	5.2
Caucasian (non-Hispanic)	6.0	4.0	3.0
Gender:			
Female	25.1	23.7	4.8
Male	11.1	3.8	9.3
Total	18.7	14.5	6.9

NOTE.—DHS = Department of Homeless Services; ACS = the New York City Administration for Children's Services. All relationships are statistically significant for χ^2 test ($p < .001$).

* "Either shelter system" category reflects the unduplicated sum of the other two columns.

Stub column (the left-most column in the table)

Entries should be as brief as possible. Stub column entries are often layered (see example A). Note the use of colons and indentations to differentiate the various layers. Stub column entries and subentries should

be confined to one column. Data in columns to the right of the stub column should be in the same row as the stub column entry. Stub column entries should not run across into the body of the table. If necessary, the entries are broken and runover lines are indented.

Cut-in heads

Cut-in heads are used when the contents of columns change-that is, when new labels are needed for the data in the columns. This is the only circumstance in which headings should appear in the body of the table. See example C below for the correct use of cut-in heads.

Example C

Table 1

TRENDS IN JOINT CUSTODY, CHILD SUPPORT PAYMENTS, AND VISITATION

	YEAR DIVORCED			
	1981–84	1985–88	1989–92	1993–98
Legal custody status (%):				
Joint custody	20.9	27.4	31.2	34.2
Mother, sole custody	66.3	60.7	57.3	53.5
Father, sole custody	12.8	11.9	11.5	12.2
	SURVEY YEAR			
	1992	1994	1996	1998
Child support payments (real 1997 $):				
Joint custody	2,390	2,526	2,656	2,753
Mother, sole custody	1,956	1,994	1,989	1,990
Father, sole custody	401	826	952	667
Visitation (days with non-resident parent):				
Joint custody	80.4	74.4	70.8	85.8
Mother, sole custody	34.1	34.4	37.2	39.1
Father, sole custody	45.4	48.8	46.4	56.3

NOTE.—1992–98 CPS-CSS. Sample limits to parents ages 15–65.

Rules are used above and below cut-in heads. Note that these rules cover only the columns that change. They do NOT extend into the stub column. As with headings at the top of a table, cut-in heads may have more than one level.

Total rule

A total rule is to be used only when there is a total that is actually the SUM of the numbers in a column. It is not to be used for averages, means, or other numbers that may summarize the data in a column, but that are not totals. See example B. The total rule extends only across columns with totals. It should not extend into other columns that do not have totals. Missing data: Three centered ellipses may be used in blank cells; it is also acceptable to leave these cells empty.

Body of the table

Wherever possible, the body of the table should be free of symbols (%, $, etc.) or measurement abbreviations (cm, kg, etc.). Symbols should appear in the column head when they apply to all values in the column, or in the stub column when they apply to all values in the row. Sometimes they can even be placed at the end of the table title, if they apply to all of the data within the table.

Footnotes to tables

All notes end with a period, even if they are not complete sentences. Table note callouts should be arranged in the body of the table from left to right, top to bottom, as if reading a text. Every callout in the table should have a corresponding footnote. The type of footnote symbols used (numbers, letters, symbols) depends on the style of the journal. See individual journals' "Information for Contributors" for specific instructions. Many tables will have a general explanatory "note" containing useful information about the table (definitions of abbreviations used, etc.). This note is not numbered. It is simply labeled "note" and appears at the bottom of the table before any other specific, numbered notes. The word "note" is singular, even when there are multiple pieces of information in the same note. See example D below.

Example D

Table 5

ESTIMATES OF THE RELATIONSHIP BETWEEN MATERNAL WORK HOURS AND THE PROBABILITY IN SELF-CARE, NONLINEAR SPECIFICATION OF WORK HOURS: LOW-SES MOTHERS

MATERNAL WORK HOURS (per Week)	LOW EDUCATION		LOW INCOME	
	Logit	FE Logit	Logit	FE Logit
At most 10	−.272	−.112	.102	.194
	(.324)	(.597)	(.436)	(.821)
More than 10 and at most 20	.300	.225	.553*	.162
	(.189)	(.335)	(.238)	(.437)
More than 20 and at most 30	.618**	.199	.423*	.086
	(.158)	(.291)	(.215)	(.384)
More than 30 hours	1.358**	1.011**	1.309**	.897**
	(.086)	(.190)	(.104)	(.231)
	[.197]	[.057]	[.178]	[.108]
N	6,059	1,383	4,020	873

SOURCE.—Author's calculations using the National Longitudinal Survey of Youth, 1979 cohort.

NOTE.—AFQT = Armed Forces Qualification Test; FE = fixed effects; SES = socioeconomic status. Huber-White standard errors are reported in parentheses, and they are corrected to account for the intraindividual correlation; marginal effects for statistically significant maternal work hours coefficients reported in brackets; mothers in the low-education subsample had less than or equal to a high school education in the first year their adolescent was observed; mothers in the poor subsample had family incomes below the poverty threshold in either 1985, 1986, or 1987. All models contain controls for the adolescent's age, the mother's highest grade completed, the mother's marital status, the number of adults and the number of children in the mother's household, and the year; the logit models also include controls for the adolescent's sex, African American, Hispanic, the mother's AFQT score, if the mother has traditional values, and the mother's age when she had her first child.

* $p < .05$.

** $p < .01$.

Some tables also have an unnumbered note labeled "sources" (or "source" if only one source is cited). This is used to list the sources that were used in constructing the table. If such a note is used, it should precede all other footnotes, including the "general" note. See example D above. Footnotes indicating standard levels of significance in statistical tables usually use asterisks. These footnotes should follow all other footnotes at the bottom of the table. See example D.

Trends in Ecology and Evolution

Require a short, explanatory title.

Abbreviations (if not defined in the main text) and full explanations should be footnoted (using superscript letters)
Use Word table template for tables and submit as part of the main document (not as separate file). Do not insert tables as images.
Ensure that tables are cited in the main text.
References cited in tables should be in a separate column and listed in the main reference list (in sequence from end of main reference list).
A maximum of five elements per article (i.e. Figures, Tables or Text Boxes)

Zoological Journal of the Linnean Society

Keep these as simple as possible, with few horizontal and, preferably, no vertical rules. When assembling complex tables and data matrices, bear the dimensions of the printed page (225 × 168 mm) in mind; reducing typesize to accommodate a multiplicity of columns will affect legibility.
